# Pain modulation by intranasal oxytocin and emotional picture viewing — a randomized double-blind fMRI study

**DOI:** 10.1038/srep31606

**Published:** 2016-08-22

**Authors:** Matthias Zunhammer, Sandra Geis, Volker Busch, Peter Eichhammer, Mark W. Greenlee

**Affiliations:** 1 Klinik für Neurologie, Universitätsklinikum Essen, Hufelandstraße 55, 45147 Essen, Germany; 2 Lehrstuhl für Experimentelle Psychologie, Universität Regensburg, Universitätsstrasse 31, 93053 Regensburg, Germany; 3 Klinik für Psychiatrie und Psychotherapie, Universität Regensburg, Universitätsstraße 84, 93053 Regensburg, Germany

## Abstract

The hormone oxytocin has been hypothesized to influence the emotional dimension of pain. This randomized, placebo-controlled, double-blind, crossover study explored whether intranasal oxytocin and emotional context can affect heat pain perception in 30 healthy male volunteers. After receiving 36 IU oxytocin or placebo, participants underwent functional Magnetic Resonance Imaging (fMRI) during which noxious and non-noxious thermode heat stimuli were applied. Simultaneously, scenes from the International Affective Pictures System (IAPS) with positive, neutral, and negative emotional valence were shown. Heat intensity and unpleasantness ratings were obtained. The activity of whole-brain correlates of heat processing was quantified via multi-voxel pattern analysis. We observed no appreciable main effects of oxytocin on ratings or neural pain correlates. Effects of emotional picture valence on ratings were smaller than reported in previous studies. Nevertheless, oxytocin was found to significantly enhance the influence of picture valence on unpleasantness ratings at noxious heat levels. No corresponding changes in whole-brain correlates of heat intensity processing were found. Our study provides evidence that intranasal oxytocin increases the effects of emotional context on the subjective unpleasantness of experimental heat pain. Future studies are needed to determine whether this effect can be utilized in clinical settings.

Oxytocin is a mammalian neuropeptide known for its role in social and affective processing; it is thought to have mild anxiolytic effects^1^ and to improve stress coping, especially in social situations^2^,^3^. The application of oxytocin via nasal spray has been suggested to elevate central nervous oxytocin levels^4^,^5^,^6^ and shown to have a benign side-effect profile^7^. A number of animal studies indicate that oxytocin may have anti-nociceptive properties (for review see ref. 8). Human studies have yielded mixed results: Rash and Campbell found that a single dose of intranasal oxytocin reduced pain and associated heart rate accelerations in a cold-pressor model^9^. Kessner *et al*.^10^ found that oxytocin increases placebo-analgesia in an experimental placebo-paradigm, but did not observe effects on heat pain ratings per se^10^. Goodin *et al*.^11^ showed that oxytocin increased the efficacy of a pain-counter-pain paradigm and improved mood, but found no evidence for oxytocin effects on ischemic, or pressure pain^11^. Singer *et al*. found no effect of oxytocin on empathy and unpleasantness of electric stimulation in males^12^. Recently, we showed that oxytocin decreased heat intensity ratings, but these changes had low effect sizes and were present across noxious and non-noxious temperatures^13^. Interestingly, oxytocin has been found to alter the processing of emotional pictures^14^. Rash and Campbell^9^ hypothesized accordingly, that the observed anti-nociceptive effects of intranasal oxytocin may be mediated by “mitigating negative mood”, implying that oxytocin affects pain processing via affective networks.

Emotionally laden stimuli^15^,^16^,^17^, such as emotional scenes taken from the international affective picture system (IAPS)^15^,^18^,^19^ have repeatedly been shown to modulate experimental pain ratings and physiological correlates of pain. Commonly, stimuli associated with negative emotional valence have been found to increase, whereas stimuli with positive valence have been found to decrease experimental pain (for review, see ref. 20). While fMRI studies have identified correlates of emotional pain modulation within several brain regions associated with pain^21^,^22^, it is unclear whether emotional context increases or decreases the sensory processing of noxious stimuli on whole brain level. Yet, the advent of predictive machine-learning techniques in functional imaging of pain^23^,^24^,^25^,^26^ has provided us with the means to estimate, whether potential pain modulators, such as emotional context and oxytocin, affect pain processing networks as a whole.

Oxytocin has been suggested as a candidate drug for pain management^8^, however, the mixed results of human studies^9^,^10^,^11^,^12^,^13^ highlight that a better understanding of the role of oxytocin in human pain is needed. Given that oxytocin has been found to affect the processing of emotional stimuli^14^,^27^,^28^,^29^, we hypothesized that a single-dose of intranasal oxytocin may alter behavioural and cerebral responses to pain depending on emotional context.

## Aims

In the present study we aimed at exploring the effects of intranasal oxytocin on experimental heat pain under modulation by emotional picture viewing. Sensory and affective pain dimensions were measured by obtaining participants’ heat intensity and heat unpleasantness ratings. Perceived heat intensity was further estimated quantitatively from fMRI images using multi-voxel-pattern analysis (MVPA).

## Methods

### Study design

In this exploratory, placebo-controlled, double-blind, cross-over trial, 36 participants were recruited and randomly assigned to Group A or B. A fully random allocation sequence was generated by the Center for Clinical Trials at the University of Regensburg. According to this sequence, the Hospital Pharmacy of the University of Erlangen labeled and numbered the medication containers. At study inclusion, participants were assigned sequential participant numbers by author P.E. and hereby allocated to Group A or B at random. A participant flow-chart and a checklist according to the CONSORT criteria is available online. Participants in both groups completed an initial training visit where the testing procedures were practiced outside of the fMRI and without medication applied. Two experimental visits followed: At Visit 1 Group A received placebo and Group B received oxytocin; at Visit 2, Group A received oxytocin and Group B received placebo. A washout-period of ≥7 days between the visits was set to minimize the risk of carry-over effects. Under both medication conditions, the effects of factors heat (non-noxious, 44.7 °C and noxious, 47.1 °C) and emotional picture viewing (positive, neutral, negative) were tested during fMRI scanning. Baseline-temperature (35.0 °C) and scrambled picture conditions were assessed as additional control conditions.

### Participants and ethics statement

Healthy right-handed male volunteers between 18 to 50 years of age were recruited by advertisement at the University of Regensburg, aiming at a sample size of 36. The study sample was restricted to males due to the sex-specific nature of oxytocin^3^ and limited resources. An a-priori power analysis indicated that the sample size entailed the power to detect effect sizes previously reported by experiments using emotional pain modulation (see: Supplement 1). Exclusion criteria were any acute or chronic medical condition, as well as recent use of psychotropic or analgesic medication (details see: Supplement 2). Participants were paid a compensation of 10 Euros per hour. The study was approved by the ethics committee of the University of Regensburg (Approval Number: 11-111-0322), as well as the responsible federal medical agency. The trial has been pre-registered in the EU Clinical Trials Register (EUDRA-CT: 2009-015115-40; December 5^th^, 2011) and categorized as a Phase I trial. Since the EU Clinical Trials Register does not publish Phase I trials, the original study protocol was re-registered and made publicly available under https://osf.io/3prdj/ as of March 31^st^ 2016. Informed consents were obtained from all participants and the study was performed in accordance to the Declaration of Helsinki.

### Procedures

**Figure 1:**
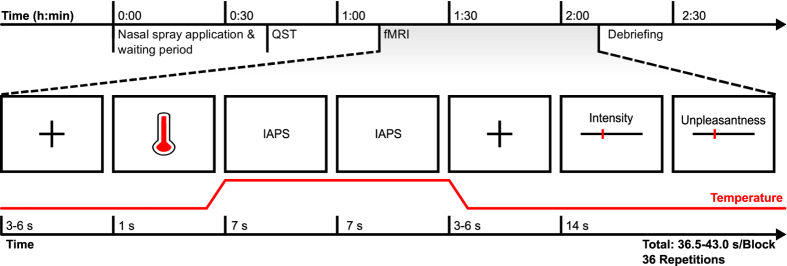
Study procedures. The upper row shows the schedule of study procedures, the lower row shows one example trial from the fMRI block design: After a fixation period and a cue, heat stimuli with three different temperatures were applied: 35.0 °C (baseline), 44.7 °C (hot, not painful), or 47.1 °C (hot, painful). Two IAPS pictures were shown during thermal stimulus plateau. Pictures either showed scenes with “positive”, “negative”, or “neutral” valence, or scrambled images. Both pictures in a given trial showed the same picture condition. After thermo-visual stimulation, participants were asked to rate heat intensity and unpleasantness on two consecutive visual analogue scales. Abbreviations: fMRI: functional Magnetic Resonance Imaging, IAPS: International Affective Picture System, QST: Quantitative Sensory Testing.

An overview on the experimental procedures is provided in Fig. 1 (upper row). Each participant received 32 IU of oxytocin, or placebo (Syntocinon Spray, Sigma Tau, Rome, Italy). The placebo spray was identical with the verum spray but contained no oxytocin. Participants self-administered four spray puffs per nostril under supervision and according to recommendations by Guastella *et al*.^30^. After intranasal application, participants were given a resting interval of 40 minutes. Then, quantitative sensory testing (QST)^31^ was performed for 20 minutes. Finally, two fMRI experiments followed in pseudo-randomized order. The QST and one fMRI experiment were part of a parallel study and are published in Zunhammer *et al*.^13^. The present study and its conclusions are based on the results of the remaining fMRI experiment; imaging data from the parallel study were used as training data for MVPA.

For the present fMRI experiment, a factorial block design with 36 trials and a total duration of 21.9 to 25.8 minutes was selected; a typical block is outlined in Fig. 1 (lower row). Each trial started with a visual cue (1 s). After a 0.5 s fixation period, thermode temperature either rapidly increased (10 °C/s) to 44.7 °C (hot, non-painful), or 47.1 °C (hot, painful), or remained at 35.0 °C (baseline control). Each target temperature was kept constant for 14 s (plateau phase), before ramp-down to baseline (10 °C/s).

Two pictures were shown during each plateau phase, the first picture started with the onset of the plateau temperature, the second followed after 7 seconds and ended with the end of the plateau temperature. In any given trial, both pictures were from the same condition, so either “negative”, or “neutral”, or “positive”, or “scrambled”. Pictures were drawn randomly and without repetitions. No picture was shown twice over the course of the experiment. Each combination of temperatures (35.0 °C, 44.7 °C, 47 °C) and pictures (negative, neutral, positive, scrambled) was repeated three times per session. The sequence of conditions was pseudo-randomized to avoid clustering.

Each thermo-visual stimulus pair was followed by 3 to 6 s of rest. Then, participants were asked to rate the perceived heat “intensity” and “unpleasantness” on two consecutive VASs, with endpoints labelled “no stimulus perceived”/“not unpleasant” and “maximally intense”/“maximally unpleasant”, respectively. Participants were instructed carefully how to rate and distinguish heat intensity and unpleasantness using standardized instructions based on Dannecker *et al*.^32^; the original instructions (in German) are provided as Supplement 3. In addition, participants were asked to rate whether the stimulus was painful, or not. Participants were required to submit all three ratings within 14 s via button press otherwise the next trial ensued. When ratings were submitted in less than 14 s, a fixation cross was displayed for the remaining time. The next block followed after another 3 to 6 s fixation interval. Participants therefore could not influence the total duration of the experiment. Two training blocks at 44.7 and 47.1 °C were conducted before the beginning of each session to reduce novelty effects and to pre-condition the skin patch. Presentation 14.9 for Windows (Neurobehavioral Systems Inc., USA) was used to coordinate image acquisition and stimulation.

### Thermal and visual stimulation

Heat stimuli were applied using a MR-safe 30 × 30 mm thermode attached to a Thermosensory Analyzer II (Medoc, Israel). The thermode was attached to the left volar surface of the lower arm, 10 cm or 15 cm proximal from the wrist crease, by using an elastic strap. The stimulated locations were switched in-between fMRI runs in random order. The non-noxious and noxious temperature levels of 44.7 and 47.1°C were determined in a pilot experiment.

Visual stimuli were presented on a screen attached to the head-end of the MR-coil, with a resolution of 1024 × 768 pixels and with a frame-rate of 60 Hz. Participants could see the full display via a mirror attached to the head coil. For emotional stimulation, three picture sets were composed from the IAPS database^33^ (for details see: Supplement 4). Each set comprised 36 pictures (18 for each study visit) with “positive”, “neutral”, or “negative” emotional valence. Positive and negative sets were matched with respect to emotional arousal. Positive, neutral and negative sets were matched with respect to the number of social (scenes depicting humans) and non-social (landscapes, objects, animals) scenes. A “scrambled” picture set was created by applying Fourier-transformation^34^ on pictures randomly drawn from the three previous sets. Scrambled picture viewing was included as an additional control condition to explore whether oxytocin affects picture processing per se. All picture sets were matched with respect to luminance.

### fMRI: General information

MR-image acquisition was performed using a 3-Tesla Allegra Head Scanner (Siemens, Germany) equipped with a single channel head coil. Each session, between 662 and 779 functional brain volumes were obtained with a T2*-weighted Echo-Planar Imaging sequence (TR = 2000 ms, TE = 30 ms, interleaved slicing, flip angle = 90°, 3 × 3 × 3.5 mm voxel size, including a 16% slice-gap, FoV = 192 × 192 mm), covering the full brain in 34 horizontal slices co-planar to the anterior and posterior commissure. A high-resolution structural head volume with 160 sagittal slices was obtained, using a Magnetization Prepared Rapid Gradient Echo (MP-RAGE) sequence (TR = 2250 ms, TE = 2.6 ms, flip angle = 9°, 1 × 1 × 1 mm voxel size, FoV = 256 × 256 mm).

### fMRI: Image pre-processing

Image pre-processing was performed using SPM 8. The first five volumes of each run were discarded to account for T1-saturation effects. Image volumes were slice-timing corrected using the middle slice as a reference. Volume-time series were realigned and re-sliced to the first scan to account for head motion, using SPM’s rigid body transformation with 4^th^ degree B-spline interpolation. Anatomical volumes were co-registered to the mean functional volume and segmented using SPM 8’s tissue probability maps. The realigned functional volumes were normalized to MNI space (2 × 2 × 2 mm voxel size) using the segmentation parameters. A Gaussian full-width at half maximum (FWHM) kernel of 8 mm was applied. Extra-cerebral regions were excluded from analysis using SPM’s brain-mask.

### FMRI: first-level analysis

For each participant SPM’s univariate linear model approach was used to obtain first-level beta maps^35^. Each of the 24 experimental conditions (2 medication × 3 temperature × 4 picture-sets) was modelled as a boxcar-predictor, the “on” phase corresponding to the 14-second plateau of heat stimulation with a delay of 5 s to account for the known onset- and cessation-latency of thermode heat perception^36^. The rating periods, as well as the six parameters of head-motion estimated by SPM’s realignment function were modelled as nuisance regressors. Predictors were convolved with SPM’s canonical hemodynamic response function and a temporal high-pass filter (width: 400 s) was applied. SPM’s autoregressive (AR1) co-variance matrix was used to account for serial correlations. First level analysis yielded 24 beta-maps per participant, each representing the individual pattern of whole brain BOLD-signal change observed for a given experimental condition.

### FMRI analysis: pattern training

MVPA was performed following the approach described by Wager *et al*.^23^, details are provided as Supplement 5. In short, a multi-voxel weight map was trained and cross-validated using data from the published parallel experiment^13^. The parallel experiment matched the present in most design parameters, with the difference that there were eight, instead of three temperature levels and no visual stimuli. We aimed at predicting individually perceived physical stimulus intensity from first-level beta-maps. Heat temperature was defined as the prediction target and the first-level beta maps representing the eight temperature levels were defined as predictive features. The first-level beta maps were z-transformed by-voxel and underwent dimensionality reduction via principal component analysis (PCA). The PCA reduced the number of candidate features from 318677 voxels to 469 principal components, retaining 99.9% of variance. The 469 principal components were then used as linear predictors in a simple regression model. A least-squares procedure with elastic-net regularization (*lasso* function, Statistics and Machine Learning Toolbox, MATLAB 2014b) was employed to estimate regression weights. Leave-one-subject-out cross-validation (LOSO-CV) was performed to estimate the performance of the regression-weights on novel datasets. The regularization parameter lambda and the shrinkage-parameter alpha were adjusted in order to minimize LOSO-CV-error and to optimize predictor sparsity. The obtained PCA-beta-weights were back-projected to voxel space using the original PCA-coefficients.

### FMRI analysis: applying the pattern

**Figure 2:**
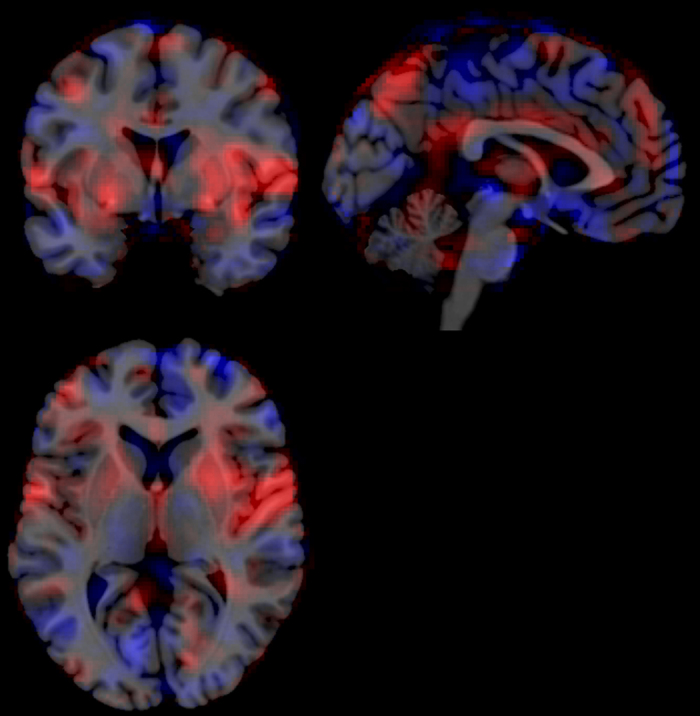
Multivoxel pattern for the prediction of noxious heat temperatures. An illustration of the predictive linear regression coefficients used to predict noxious heat temperatures from whole-brain fMRI images. The weight maps were trained and cross-validated with data from the parallel experiment, using a LASSO-PCA^43^ approach. Red voxels denote positive predictive weights and blue voxels negative predictive weights; the weight magnitude is implied by colour saturation (range: 8.14*10^−4^ to −5.35*10^−4^, arbitrary units). Signal increases in red areas and signal decreased in blue areas both result in increased multi-voxel heat estimates. Areas with high saturation contribute strongly to the prediction, whereas areas with low saturation have little influence. Slices are taken at MNI coordinates (x = −2, y = 2, z = 5).

An illustration of the multi-voxel weights map is provided in Fig. 2. The mask was applied on all first-level beta-images from the present experiment, by calculating the by-voxel dot product, yielding one multi-voxel heat estimate (MHE) per participant and experimental condition.

### Statistics

Analyses were performed with *R* (v3.2.2). Ratings and MHE with a probability of less than 1:5000 on the two-tailed normal distribution were defined as outliers on a within-subject-within-temperature basis. Ratings were aggregated across repetitions to obtain one data-point per subject and experimental condition to match the number of SPM’s beta images. The linear mixed model function “lmer” (lme4 v1.1-9)^37^ was used to estimate the effects of medication (placebo, oxytocin), heat (non-noxious, noxious), and emotional picture valence (negative, neutral, positive) on the mean-centred and standardized outcome measures. All factors and interactions were modelled as fixed effects. Within-participant dependencies were modelled as by-subject random intercepts and by-subject random slopes for all effects, aiming for a maximal random effects structure^38^. An unstructured covariance matrix was used. Conclusions are based on Type-III ANOVAs (lmerTest v2.0-29), Kenward-Roger approximation of degrees of freedom) and estimated marginal mean effects with 95% Confidence Intervals (CI, see ref. 39). Pairwise estimated marginal means were obtained with lsmeans (v2.20-23). Effect sizes are provided in unstandardized and standardized (β) form. Control analyses were performed for the scrambled picture viewing and baseline temperature (35.0°C) conditions, as well as categorical pain ratings. The full statistical R syntax is provided as Supplement 6.

## Results

### Sample description and dataset

Thirty participants completed the study and were eligible for analysis. Mean age at study inclusion was 24.9 years (range: 19 to 30). Participants were allocated to receive oxytocin in the first session in 53% of cases. Further sample details and details on the six excluded participants are provided in Zunhammer *et al*.^13^. The last three trials of one participant (both sessions) were lost to technical failure. Participants completed their ratings within a mean of 7.5 s, 95% CI [7.4, 7.6]. Eight ratings were not submitted within 14 s and excluded. The outlier criterion was fulfilled by 0.8% of intensity ratings, 0.8% of unpleasantness ratings, and one MHE value; these data were excluded.

### Intensity and unpleasantness ratings

**Figure 3:**
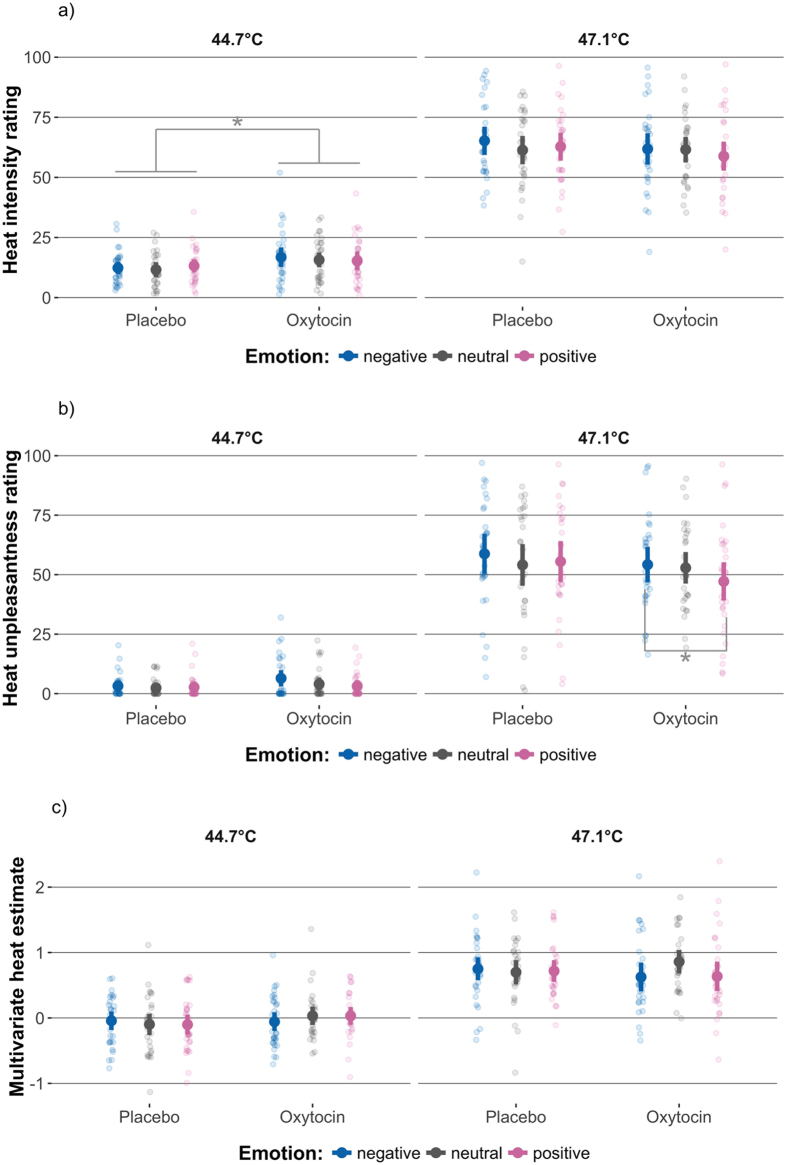
Ratings and multi-voxel heat estimates. Effects of oxytocin (placebo vs. oxytocin), temperature (44.7 vs. 47.1 °C), and emotional picture valence (negative vs. neutral vs. positive) on (**a**) intensity ratings, (**b**) unpleasantness ratings, and (**c**) multi-voxel heat estimates (MHE). Figures depict the marginal means and 95% Confidence Intervals (CI) estimated by a mixed model analysis, plotted on top of single subject data. Asterisks indicate significant contrasts (p < 0.05) in the follow-up of interactions involving oxytocin.

The results of the mixed-model ANOVA for ratings are listed in Table 1. As expected, there was a significant main effect of temperature on heat intensity ratings (Table 1, Fig. 3a); noxious, compared to non-noxious heat increased intensity ratings by a mean of 47.8 points VAS (95% CI [43.3, 52.2], β = 1.75) across conditions. Further, there was a non-significant tendency towards an effect of emotional picture valence (Table 1, Fig. 3a); negative pictures tended to increase heat intensity ratings compared to neutral (1.51 points VAS, 95% CI [0.12, 3.15], β = 0.06) and positive (1.53 points VAS, 95% CI [0.10, 2.96], β = 0.06) pictures across conditions. The ANOVA further indicated a significant temperature-dependent effect of oxytocin (Table 1, Fig. 3a); at non-noxious heat levels oxytocin increased intensity ratings by a mean of 3.54 points VAS (95% CI [0.88, 6.20], β = 0.13) across all levels of emotional valence. At noxious heat levels, oxytocin decreased intensity ratings, but by a smaller, more variable amount (−2.38 points VAS, 95% CI [−7.8, 3.07], β = −0.09).

There was a main effect of temperature on unpleasantness ratings (Table 1, Fig. 3b); noxious, compared to non-noxious heat increased unpleasantness ratings by a mean of 50.1 points (95% CI [43.6, 56.6], β = 1.68) across all conditions. Further, there was a significant main effect of picture valence; viewing negative compared to neutral (+2.31 points VAS, 95% CI [0.43, 4.19], β = 0.08) and positive pictures (+3.55 points VAS, 95% CI [1.60, 5.50], β = 0.12) increased heat unpleasantness ratings across all conditions, whereas positive, compared to neutral picture viewing tended to decrease unpleasantness ratings (−1.24 points VAS, 95% CI [−0.56, 3.03], β = 0.04).

There was a significant 3-way interaction effect of oxytocin, heat and emotional valence on unpleasantness ratings (Table 1, Fig. 3b). The interaction effect was driven by increased differences between the positive and negative picture conditions under oxytocin, at noxious heat levels (−7.08 points VAS, 95% CI [−11.1, −3.07], β = −0.24). The corresponding differences under placebo were about half that size (−3.26 points VAS, [−7.50 0.99], β = −0.11). In summary, these results replicated the known effects of emotional picture valence on unpleasantness ratings^15^,^18^,^19^,^21^,^40^,^41^,^42^ and suggest that oxytocin augments these modulatory effects at noxious heat levels.

### MVPA estimates of heat processing

We trained linear regression weights in order to predict the applied thermode heat intensity from fMRI images using independent fMRI runs from the same participants^13^. A description of cross-validation results for the multi-voxel weights mask is provided in Supplement 5. Applying the mask to our present fMRI dataset, we found that the resulting multi-voxel heat estimates (MHE) correlated with heat level (r = 0.62, 95% CI [0.57, 0.66]), as well as intensity (r = 0.64, 95% CI [0.60, 0.68]) and unpleasantness ratings (r = 0.61, 95% CI [0.56, 0.66]). To further illustrate the performance of our weight mask in terms of classification performance, we dichotomized MHE scores, as well as intensity and unpleasantness ratings with a median split: Median-split MHE correctly discriminated noxious (47.1 °C) from non-noxious (44.7 °C) heat levels in 76.3% of cases (precision: 61.5%). Median-split intensity and unpleasantness ratings correctly predicted heat level in 73.5% (precision: 67.2%) and 85.3% (precision: 74.1%) of cases, respectively. These figures suggest that MHE had an acceptable predictive validity and that its reliability was somewhat lower than that of participant ratings.

Accordingly, the mixed-model ANOVA for MHE estimates indicated a significant main effect of temperature (Table 1, Fig. 3c). Heat temperature increases from non-noxious to noxious levels (+2.4 °C) increased MHE by +0.75 units, 95% CI [0.64, 0.87] (β = 1.26), which corresponds to a large effect size. No significant main effects of oxytocin or picture valence and no significant interaction effects were found. Oxytocin showed a non-significant tendency to decrease MHE’s, when participants viewed negative (−0.16 units [−0.28, −0.05], β = −0.27), or positive (−0.11 units [−0.24, 0.01], β = −0.19) compared to the neutral pictures, whereas opposite effects were observed under placebo conditions (negative-neutral: 0.05 units, [−0.10, 0.21], β = 0.09; positive-neutral: 0.01 units, [−0.10, 0.12], β = 0.01). These results corroborate the behavioural findings by suggesting that oxytocin augmented the effects of emotional picture viewing on cerebral processing of noxious heat. However, oxytocin and negative picture viewing tended to *decrease* MHE, whereas negative picture viewing tended to increase unpleasantness ratings across conditions. Therefore MHE and rating results are dissimilar and unlikely to reflect the same process.

### Additional controls: scrambled picture viewing, baseline temperature and categorical pain ratings

In order to explore potential oxytocin effects on pain modulation by visual distraction, participants were shown scrambled pictures in addition to the normal (emotional) picture sets. The analyses performed above were repeated, replacing the factor “Emotion” by the factor “Scrambled” (levels: scrambled pictures vs. pictures). In summary, viewing pictures compared to scrambled pictures slightly decreased both intensity ratings and MHE across all temperatures, whereas oxytocin showed no main or interaction effect (see: Supplement 7).

Further, we examined the effects of normal and scrambled picture viewing on MHE at baseline temperature (35.0 °C) to determine whether the MHE was sensitive to visual stimulation. Emotional picture valence and scrambled pictures had no significant effect on MHE at baseline temperature, yet, effect sizes and directions of effect were similar to what was observed at higher temperatures (see: Supplement 7). Together with the effects of temperature stimulation, these results indicate that the MHE is far more sensitive to thermal than to visual stimulation alone. No effects of oxytocin on MHE were found at baseline temperature.

Categorical pain ratings were obtained as a control measure, in order to verify that the temperature conditions chosen qualified as non-noxious and noxious, respectively. Only 3.1% of all stimuli at 44.7°C were classified as painful, whereas 83.6% of all stimuli were classified as painful at 47.1°C. Not a single baseline stimulus (35.0°C) was rated as painful (see: Supplement 7). Of note, the hot, non-noxious temperature level used in the present experiment (44.7°C) is slightly above the heat pain threshold that was determined for un-stimulated skin using the method of limits in the parallel study (44.5°C)^43^. However, temperatures of 44.7°C were perceived as non-noxious — likely due to local de-sensitizing effects induced by the pre-conditioning training stimuli and the repeated heat stimulation.

## Discussion

We found that intranasal oxytocin increases the effects of emotional valence on unpleasantness ratings at noxious heat levels (Fig. 3b). Our study therefore provides evidence that intranasal oxytocin influences emotional pain modulation in healthy men. Similar to previous studies^9^,^10^,^11^,^12^,^13^ the observed oxytocin effects were small and below the threshold of clinical significance^44^,^45^. Our results add to a growing body of evidence suggesting that intranasal oxytocin does not have strong, straightforward analgesic effects in humans^10^,^11^,^12^,^13^. Rather, the effects of oxytocin on pain seem to be subtle and dependent on emotional context.

While we found a 3-way interaction effect of oxytocin, temperature and emotional valence on unpleasantness ratings, intensity ratings merely showed a significant 2-way interaction effect of medication and temperature (Fig. 3a), but no reliable effects of emotional valence. Such results are intuitive, considering that unpleasantness ratings target affective aspects of pain by definition. Consequently, unpleasantness ratings have a higher sensitivity to detect effects of emotional pain modulation than intensity ratings^17^. Accordingly, the unique effect of emotional valence was β = 0.06 SDs for intensity and β = 0.12 SDs for unpleasantness ratings in our present study. The significant 2-way interaction effect of medication and temperature for intensity ratings may therefore represent a covert 3-way interaction that remained undetected due to a lack of sensitivity for emotional valence.

Numerous previous studies reported that emotional picture valence modulates pain ratings at noxious stimulus levels^15^,^18^,^19^,^21^,^40^,^41^,^42^. In a preliminary analysis we estimated the median standardized effect size of negative, compared to positive pictures as 0.44 SDs (see: Supplement 1). The standardized effect of emotional picture valence on rating behaviour in our present study was markedly lower. Regarding the negative pictures, this discrepancy may be explained by our strict criteria for IAPS picture selection (see: Supplement 4). We excluded displays of sharp objects, mutilation, and blood to avoid the possibility that imagination of pain confounded negative affect^46^,^47^. However, the effects of positive, compared to neutral pictures were also lower than expected. The fact that two pictures of similar valence were shown in immediate succession may have induced contextual interaction effects that may have diminished the perceived valence of the second picture shown. Aside from peculiarities of our image set and experimental design, our results may also indicate that effect sizes of emotional pain modulation in the literature are inflated by publication bias^48^.

Interestingly, we found that emotional picture valence seemed to affect unpleasantness ratings across noxious *and* non-noxious temperatures (see: Fig. 3a,b). These results add support to observations by Kenntner-Mabiala *et al*.^49^,^50^, who showed that emotional pictures affect participants’ responses at noxious and non-noxious stimulus levels^49^,^50^. Together, these findings indicate that experimental emotional context may not affect pain in an exclusive manner. Emotional valence may modulate non-noxious and noxious stimulus intensity ratings by common mechanisms. For one, the changes in ratings at non-noxious stimulus levels may represent baseline adaptions in ‘‘preparedness” to threats with the ultimate purpose to increase the probability of successful evasive responses — a view compatible with the motivational priming hypothesis^51^. Likewise, “good participant behaviour” and social desirability may partly explain the effects of emotional pictures on ratings, since picture valence and the expected direction of effect are obvious for the subjects.

Using functional neuroimages from the parallel experiment^13^, we trained and cross-validated a multi-voxel pattern mask (see: Fig. 2) that allows to estimate physical heat intensity from novel fMRI images. The mask can be seen as a whole-brain summary of positive and negative associations between BOLD signal changes and physical heat intensity, whereas each voxel is weighed according to its association strength and according to the reliability of its information content across subjects. Unsurprisingly, the mask shows strong correspondences with previous pain imaging results, e.g. voxels in the insular and cingulate cortices show strongly positive weights (compare: Fig. 2 and ref. 52). Yet, the purpose of our MVPA approach was not to localize, but to quantify whole-brain heat pain processing and modulation. By applying the mask to fMRI volumes from our present experiment via matrix multiplication, MHE values were obtained, which represent the degree of similarity between the fMRI volumes and the functionally defined heat intensity pattern.

The MHEs for our present fMRI dataset (see: Fig. 3c) could predict noxious and non-noxious heat levels with an accuracy comparable to participants’ intensity ratings. The reliability of these predictions was somewhat lower. MHEs further showed moderate associations with intensity and unpleasantness ratings, indicating its concurrent validity as a non-behavioural measure of heat intensity perception. Emotional visual content had minor effects on MHEs at baseline temperature conditions confirming that the MHEs primarily reflect somatosensory processes (see: Supplement 7). Moreover our MVPA approach was sensitive to detect certain forms of pain modulation: when participants watched intact, compared to scrambled pictures, participants’ intensity ratings decreased across temperatures. We found that MHEs paralleled these decreases (see: Supplement 7), indicating that our MVPA mask could detect the distracting effects of representational picture viewing on thermoception^53^.

Nevertheless, we could not find significant main or interaction effects of oxytocin or emotional picture valence with on MHEs. If anything, positive and negative pictures tended to decrease MHEs under oxytocin conditions (Fig. 3c). This tendency somewhat corroborates the behavioural findings by showing that oxytocin had *some* effect on cerebral processing. However, the fact that negative picture viewing tended to reduce MHE and to increase unpleasantness ratings indicates that our mask did not mirror the observed behavioural effects of emotional valence. We offer two straightforward explanations for these results: Firstly, the changes in whole-brain heat processing (MHE) due to oxytocin and emotional picture viewing may be too small to be captured by our MHE. Secondly, the behavioural effects of oxytocin and emotional picture viewing may be encoded in confined sub-portions of the heat-intensity mask, or be represented by entirely different networks, e.g. networks related to threat evaluation^28^, or the evaluation of hedonic value^27^.

Like all studies on behavioural effects of intranasal oxytocin, our study is limited by uncertainties regarding oxytocin administration. A few studies are now available that allow inferences on the time-course and distribution of oxytocin in the human CNS after intranasal application: A small study in healthy volunteers found that intranasal oxytocin did not lead to increased CSF levels of oxytocin until 75 minutes after administration^5^. Another study found that perfusion-changes in limbic brain regions “showed a peak response 39–51 min after IN-OT [intranasal oxytocin administration], followed by a gradual diminution of effects”^54^. These studies were not available at the time when the present study was planned. Our fMRI session took place between 70 and 130 min after nasal spray administration, which is considerably later than most previous studies^14^,^29^,^55^,^56^ and later than the time-windows captured by studies on cerebral pharmacokinetics of oxytocin^5^,^54^. Therefore, the oxytocin effects observed in our present study may be limited to the time frame of measurements and the chosen dose (32 IU). Particularly, it is possible that oxytocin effects were underestimated since the measurements were taken after oxytocin peaked in the brain.

Despite a-priori power analysis (see: Supplement 1), the power of our present study to detect oxytocin effects on emotional pain modulation may have been limited by the unexpectedly weak impact of emotional picture viewing on rating behaviour. Due to the sex-specific nature of oxytocin^3^ the study sample was restricted to males and therefore only allows valid generalization to the healthy, young, male population.

## Conclusion

The present study provides evidence that intranasal oxytocin increases the effects of emotional picture valence on subjective measures of heat pain unpleasantness. Together with previous findings, our results suggest that the effects of oxytocin on experimental pain and its neural correlates depend on experimental context and may be driven by participants’ cognitions and emotions^10^. Clinical studies are needed to determine whether these effects can be utilized in the treatment or prevention of chronic pain conditions. No effects of oxytocin and emotional picture viewing on whole-brain correlates of heat pain intensity were found. Sub-networks of pain processing, or networks secondary to pain processing, such the reward system or anxiety-related networks^2^, may mediate these observed behavioural effects of oxytocin and emotional picture valence.

## Additional Information

**How to cite this article**: Zunhammer, M. *et al*. Pain modulation by intranasal oxytocin and emotional picture viewing — a randomized double-blind fMRI study. *Sci. Rep.* **6**, 31606; doi: 10.1038/srep31606 (2016).

## Supplementary Material

Supplementary Information

## Figures and Tables

**Table 1:** ANOVA results.

Mixed-Model ANOVA Results		Heat Intensity		Heat Unpleasantness		Multi-voxel heat estimate	
Model Term	df1/2	F	p	F	p	F	p
Oxytocin	1/29	0.12	0.745	0.48	0.493	0.22	0.640
Temperature	1/29	**476.8**	**<0.001**	**251.8**	**<0.001**	**172.8**	**<0.001**
Valence	2/28	2.52	0.098	**6.80**	**0.004**	0.96	0.395
Oxytocin*Temperature	1/29	**5.87**	**0.022**	3.51	0.071	0.87	0.358
Oxytocin*Valence	2/28	1.25	0.303	2.32	0.116	2.87	0.073
Temperature *Valence	2/28	1.06	0.361	1.42	0.258	0.63	0.541
3-way Interaction	2/58	1.34	0.269	**3.40**	**0.040**	1.73	0.187

Type III ANOVAs were performed on mixed models using the Kenward-Roger approximation for degrees of freedom. The degrees of freedom listed under df1/2 were rounded to the next integer.
