# Supplementary material for: Pain modulation by intranasal oxytocin and emotional picture viewing — a randomized double-blind fMRI study
Source: Sci Rep. 2016 Aug 22;6:31606. doi: 10.1038/srep31606 (PMC4992880; doi:10.1038/srep31606)
Supplement: Supplementary Information [file srep31606-s1.pdf]

Supplementary Material:

Pain modulation by intranasal oxytocin and emotional picture viewing — a randomized double-blind fMRI study

Authors: Matthias Zunhammer, Sandra Geis, Volker Busch, Peter Eichhammer, Mark W. Greenlee

Supplement 1: Power analysis for intensity and unpleasantness ratings effects

Table S1.1: Experimental design

Factor	Levels	Level Names
Oxytocin	2	Oxytocin, placebo
Temperature†	2	44.7 °C, 47.1 °C
Pictures	3	negative, neutral, positive

Power analysis was performed for a repeated-measures ANOVA (assuming sphericity) by calculating the non-centrality parameter lambda [7] and determining the corresponding power with G*Power 3.1. For the sake of simplicity we planned aggregating across repetitions instead of using repetition as an additional factor.

$$\lambda = (a*b*c)/(1-r) * N * \text{Cohen's } f^2$$

Whereas,

- a, b and c are the number of factor levels of oxytocin, temperature and pictures, respectively.
- r is the correlation between the levels of a within-subject factor, or interaction effect. For all calculations, we used r = 0.40, which is a conservative approximation of the actual within-subject correlations.
- N is the total sample size (N = 30)
- Cohen's f^2 is the desired effect size. Canonical values for small (0.02) and medium (0.15) effects were used [1]. In addition, the typical (i.e. median) size of effect of emotional valence on pain ratings found in previous studies (0.049, see: Table S1.2) was tested.
- Power values corresponding to the non-centrality parameters were determined using the "Generic F-Test" function of G*Power 3.1, at an alpha level of < .05.
- Results are listed in Table S1.3.

Table S1.2: An estimation of the typical effect size of negatively compared to positively valenced IAPS pictures on pain ratings

Study	Source	Approximate Cohen's d	Approximate Cohen's f^2
Rhudy, Williams, McCabe, Russell, & Maynard, 2008	Table 3—„Pain Rating"	0.123	0.004
Kamping, Bomba, Kanske, Diesch, & Flor, 2013	Fig 2 B — „healthy controls"	0.400	0.040
Horjales-Araujo et al., 2013	Results: 3.2. „jaw muscle pain HS"	0.444	0.049
Roy, Lebuis, Peretz, & Rainville, 2011	Table S1 and Table S2	0.887 and 0.436	0.197 (Experiment 1) and 0.048 (Experiment 2)
Roy, Piché, Chen, Peretz, Rainville, 2009	Results: „pain ratings and RIII Reflex"	3.48	3.028
Median		0.436	0.049

Effect sizes were estimated by computing Cohen's d for the negative compared to positive picture valence condition according to the formula: $(Mean_{negative} - Mean_{positive})/((SD_{negative} + SD_{positive})/2)$ and transformed to Cohen's f^2 using the formula $f^2 = d^2/4$. Repeated-measures were disregarded where possible (see: Dunlap, Cortina, Vaslow, & Burke, 1996).

Table S1.3: Statistical power estimates

Factor	df	power to detect medium effect	power to detect small effect	power to detect effect of emotional valence
Non-Centrality Parameter		λ = 90	λ = 12	λ =29.4
Oxytocin	df_1=1, df_2=29	>.999	.917	>.999
Temperature	df_1=1, df_2=29	>.999	.917	>.999
Pictures	df_1=2, df_2=58	>.999	.917	>.999
Oxytocin*Temperature	df_1=1, df_2=29	>.999	.917	>.999
Oxytocin*Pictures	df_1=2, df_2=58	>.999	.917	>.999
Temperature*Pictures	df_1=2, df_2=58	>.999	.917	>.999
Oxytocin*Temperature*Pictures	df_1=2, df_2=58	>.999	.917	>.999

Statistical power (1- β error) of the present study to detect effects of oxytocin, temperature, and picture viewing on heat intensity and unpleasantness ratings. The "Generic F-Test, Post-Hoc" function of G*Power 3.1 (alpha = .05) was used to determine power values corresponding to the caluclated non-centrality parameters.

Supplement 2: In-/ exclusion criteria

Table S2.1: In-/ exclusion criteria

Inclusion criteria	Right-handed<br>Male<br>Age: 18 to 50 years
Exclusion criteria	Known allergy of, or intolerance against oxytocin, chlorbutanol, glycerin, methylparaben, sorbitol, citric acid, or nasal sprays in general,<br>Past or present chronic or acute cardiac conditions (hypo-/hypertension, arrhythmias)<br>Present acute infections (common cold, flu, diarrhea, etc.)<br>Past or major internal, neurological, psychiatric, hormonal, or chronic medical conditions<br>Recent surgery<br>Recent use of illicit drugs, psychotropic, or analgesic medication<br>Disorders of pain<br>Alcohol addiction (DSM-IV)<br>Metallic implants, or any other exclusion criteria regarding MR-safety, Claustrophobia<br>Alcohol consumption within 24 hours before visits †<br>Caffeine consumption within 12 hours before visits †

All in- and exclusion criteria were re-checked at the beginning of every study visit in a medical examination including vital signs and a structured interview.

Supplement 3: Original participant instructions regarding fMRI procedures and pain ratings (German)

„Thresholding

Untere Thermodenkante immer 7 cm von der Linie des Handgelenks entfernt!
(Nach QST Manual Version 2.1 vom 08.07.2010, Lehrstuhl für Neurophysiologie, Universitätsmedizin Mannheim; Ruprecht-Karls-Universität Heidelberg)
Die Anweisungen werden nur im Vorversuch vollständig vorgelesen. In den Folgeversuchen werden nur noch die ***dick*** *gekennzeichneten Anweisungen vorgelesen.*

„In dem nachfolgenden Test werden wir mit Hilfe verschiedener Verfahren untersuchen, inwieweit Sie Temperaturveränderungen wahrnehmen. Darüber hinaus werden wir prüfen, ab wann Sie verschiedene Testreize als schmerzhaft empfinden. Diese Tests müssen immer auf dieselbe Art und Weise durchgeführt werden. Um dies zu gewährleisten, werden Ihnen die Testanweisungen vorgelesen.
Bitte fragen Sie immer sofort nach, falls Sie eine Anweisung nicht verstanden haben.
Sollten Sie einmal einen Fehler gemacht haben (zu früh gedrückt, falsche Wertung) sagen Sie bitte sofort Bescheid, denn Korrekturen der Eingabe sind ohne weiteres möglich.“
Sie können bei allen nun folgenden Messungen die Schmerzstimulation jederzeit durch Drücken der Leertaste beenden. Die Maschine besitzt außerdem eine Begrenzung der Maximal- Temperatur. D.h. die Schmerzstimulation kann zu keiner Hautschädigung führen.

Haben Sie soweit Fragen?
„Das Gerät auf Ihrer Haut kann die Haut erwärmen oder abkühlen. Zusätzlich erhalten Sie eine Stopp-Taste, mit deren Hilfe Sie jederzeit den aktuellen Test-Reiz stoppen können. Ich sage Ihnen gleich zu jedem einzelnen Test, wann Sie die Stopp-Taste betätigen sollen.

Sagen Sie jetzt bitte, ob sich das Gerät auf Ihrer Haut warm, kalt oder unbestimmt anfühlt!

CDT „Zunächst testen wir Ihre Fähigkeit, Kälte wahrzunehmen. Drücken Sie bitte sofort auf die Stopp-Taste, wenn Sie erstmals eine Abkühlung spüren. Im Anschluss

wird sich die Thermode wieder zur Ausgangstemperatur aufwärmen. Dieser Vorgang wird in wenigen Sekunden beginnen. **Zunächst ein Probedurchlauf.“**
"Dieser Vorgang wird nun vier Mal mit jeweils ca. 10s Pause wiederholt.“

WDT „Nun testen wir Ihre Fähigkeit, Wärme wahrzunehmen. **Drücken Sie bitte sofort auf die Stopp-Taste, wenn Sie erstmals eine Erwärmung spüren.** Im Anschluss wird sich die Thermode wieder zur Ausgangstemperatur abkühlen. Dieser Vorgang wird in wenigen Sekunden beginnen. **Zunächst ein Probedurchlauf.“**
"Dieser Vorgang wird nun vier Mal mit jeweils ca. 10s Pause wiederholt.“

CPT „Nun testen wir, ab wann Sie die Abkühlung der Thermode als schmerzhaft empfinden. Dazu wird Ihre Haut abgekühlt. Irgendwann wird zu der Wahrnehmung von „Kälte“ eine weitere Empfindung hinzukommen. Dabei wird der Eindruck von „Kälte“ seine Qualität verändern mit dem zusätzlichen Eindruck eines „Brennens“, „Stechens“, „Bohrens“ oder „Ziehens“. Drücken Sie bitte sofort auf die Stopp-Taste, sobald Sie eine solche Veränderung wahrnehmen. Drücken Sie NICHT erst dann auf die Stopp-Taste, wenn die Empfindung unerträglich schmerzhaft wird. Im Anschluss wird sich die Thermode wieder hin zur Ausgangstemperatur aufwärmen. Dieser Vorgang wird in wenigen Sekunden beginnen. Zunächst ein Probedurchlauf.“
Nach dem Probedurchlauf: Auf einer Skala von 0 bis 10 (wobei 0 nicht schmerzhaft und 10 so schmerzhaft wie an dieser Stelle vorstellbar bedeutet), wie stark war der Schmerz zum Zeitpunkt des Knopfdrucks?
Proband anweisen in Zukunft dann zu drücken, wenn Sie dem Schmerz eine 1 aus 10 geben würden.
(Kurzversion: „Jetzt drücken Sie bitte sofort auf die Stopp-Taste, wenn zur Wahrnehmung von „Kälte“ eine zusätzliche Empfindung eines „Brennens“, „Stechens“, „Bohrens“ oder „Ziehens“ hinzukommt. Zunächst ein Probedurchlauf.“)
"Dieser Vorgang wird nun vier Mal wiederholt mit jeweils ca. 10s Pause.“

HPT „Nun testen wir, ab wann Sie die Erwärmung der Thermode als schmerzhaft empfinden. Dazu wird Ihre Haut erwärmt. Irgendwann wird zu der Wahrnehmung von „Wärme“ oder von „Hitze“ eine weitere Empfindung hinzukommen. Dabei wird der Eindruck von „Wärme“ oder „Hitze” seine Qualität verändern mit dem zusätzlichen Eindruck zum Beispiel eines „Brennens”, „Stechens“, „Bohrens“ oder „Ziehens“. Drücken Sie bitte

sofort auf die Stopp-Taste, sobald Sie eine solche Veränderung wahrnehmen. Drücken Sie NICHT erst dann auf die Stopp-Taste, wenn die Empfindung unerträglich schmerzhaft wird. Im Anschluss wird sich die Thermode wieder hin zur Ausgangstemperatur abkühlen. Dieser Vorgang wird in wenigen Sekunden beginnen. Zunächst ein Probedurchlauf.“

Nach dem Probedurchlauf: Auf einer Skala von 0 bis 10 (wobei 0 nicht schmerzhaft und 10 so schmerzhaft wie an dieser Stelle vorstellbar bedeutet), wie stark war der Schmerz zum Zeitpunkt des Knopfdrucks?

Proband anweisen in Zukunft dann zu drücken, wenn Sie dem Schmerz eine 1 aus 10 geben würden.

(Kurzversion: „Jetzt drücken Sie bitte sofort auf die Stopp-Taste, wenn zur Wahrnehmung von „Wärme“ oder „Hitze“ eine zusätzliche Empfindung eines „Brennens“, „Stechens“, „Bohrens“ oder „Ziehens“ hinzukommt. Zunächst ein Probedurchlauf.“)

"Dieser Vorgang wird nun vier Mal wiederholt mit jeweils ca. 10s Pause.

Probandenanweisung

Tonische Hitzeschmerztestung

Untere Thermodenkante immer 12 cm von der Linie des Handgelenks entfernt!

Diese Anweisungen werden nur im Vorversuch vollständig vorgelesen. In den folgenden Versuchen werden nur noch die ***dick*** *gekennzeichneten Anweisungen vorgelesen.*

"Wir werden nun im Kernspintomographen (MRT) messen welche Gehirnareale bei Ihnen während der Schmerzverarbeitung aktiv sind.

MRT ist eine sichere Technik, die mit einem starken Magnetfeld, aber ohne ionisierende Strahlung arbeitet und bei der in über 25 Jahren Routineeinsatz in Klinik und Forschung keinerlei Nebenwirkungen bekannt geworden sind. Einzig das Einbringen magnetischer Gegenstände die sich im oder am Körper befinden stellt eine Gefahrenquelle dar. Lose magnetische Teile könnten vom Magneten angezogen und durch die Luft gewirbelt werden. Magnetische Gegenstände am Körper könnten sich erhitzen.

Bitte legen Sie alle magnetischen Gegestände ab. (Schlüssel, Geldbörse, Gürtel, Haarspangen, Haarnadeln etc.) Das Gerät ist außerdem sehr laut, weswegen Sie Ohrenstöpsel und einen Gehörschutz tragen müssen.

Die Prozedur wird insgesamt 30 Minuten ruhiges Liegen im Gerät erfordern.

In diesen 20 Minuten werden sie 24 Mal mit Hitzereizen stimuliert. Diese gleichbleibenden Hitzereize werden knapp 15 Sekunden dauern und unterschiedliche Stärken haben. Diese Stärken werden sich in zufälliger Reihenfolge abwechseln. Die Temperaturen der Reize werden zwar z.T. schmerzhaft sein, es besteht aber keinerlei Gefahr für eine dauerhafte Haut- oder Gewebeschädigung. Es ist mit einer leichten Rötung des Stimulierten Hautareals zu rechnen, die aber nach einigen Stunden vollständig abklingt

Sie erhalten einen Nottaster mit dem Sie den Versuch bei Bedarf jederzeit abbrechen können.

Gibt es soweit Fragen?

Über einen Bildschirm werden Sie während des Versuchs verschiedene Anweisungen angezeigt bekommen:

1. Die meiste Zeit werden Sie ein sog. Fixationskreuz (*vorführen*) sehen. Schauen Sie bitte stets in die Mitte des Kreuzes, (blinzeln nicht verboten!) auch während der schmerzhaften Reize! (Augen nicht zusammenkneifen).
2. Sie werden einige Sekunden vor jedem Hitzereiz durch das Wort „Hitze" auf dem Bildschirm gewarnt.
3. Nach jedem Hitzereiz wird eine erste Skala erscheinen auf der sie mit einem Schieber eintragen sollen wie intensiv der vorausgegangene Reiz als Ganzes von Ihnen empfunden wurde.

Den Schieber können Sie mit der rechten Taste nach rechts und mit der linken Taste nach links bewegen. Mit der Mitteltaste wählen Sie eine Bewertung aus und beenden die Skala. Die Bewertung auf der Skala bedeutet folgendes:

- **Ende der Skala links: Sie haben keine Veränderung gespürt, der Reiz war nicht einmal warm.**
- **Skala bis kurz vor der Mitte: Der Reiz nimmt an Intensität/Hitzegefühl zu, ist aber noch nicht Schmerzhaft. Kurz vor dem Strich bedeutet z.B. „Heiß aber gerade nicht schmerzhaft".**
- **Skala jenseits der Mitte: Der Reiz nimmt weiter an Intensität/Hitzegefühl zu und ist schmerzhaft. Kurz nach dem Strich bedeutet z.B. „Heiß und gerade eben schmerzhaft".**
- **Ende der Skala rechts: Der Reiz ist heiß und so intensiv schmerzhaft wie an dieser Stelle vorstellbar.**

- Diese Einschätzung ist rein subjektiv. Sie können dabei nichts falsch machen, solange Sie sich Mühe geben möglichst genau zu bewerten.
- **Sie haben nur 7 Sekunden Zeit die Bewertung abzugeben. Entscheiden Sie sich also schnell und trotzdem genau!**

(*vorführen*)

Gibt es soweit Fragen?

4. Nach der ersten Skala erscheint sofort eine zweite Skala. Auf dieser zweiten Skala geben Sie bitte an wie unangenehm der vorangegangene Reiz als Ganzes von Ihnen empfunden wurde. Der Schieber wird genauso bedient wie der der ersten Skala.

Die Bewertung auf der Skala bedeutet dieses Mal folgendes:

- **Ende der Skala links: Der Reiz war nicht im Geringsten unangenehm.**
- **Ende der Skala rechts: Der Reiz war so unangenehm wie an dieser Stelle vorstellbar.**
- Diese Einschätzung ist rein subjektiv. Sie können dabei nichts falsch machen, solange Sie sich Mühe geben möglichst genau zu bewerten.
- **Sie haben wieder nur 7 Sekunden Zeit die Bewertung abzugeben. Entscheiden Sie sich also schnell und trotzdem genau!**

Bei beiden Skalen gilt: Sie müssen den Schieber zumindest einmal verschieben bevor sie die Bestätigen-Taste drücken können!

(*vorführen*)

Nach der zweiten Skala folgt nach wenigen Sekunden Fixationskreuz der nächste Reiz.

Gibt es soweit Fragen?

Weitere Anmerkungen:

1. **Sie werden eventuell das Gefühl haben, dass sich ihre Schmerzwahrnehmung nach wiederholter Stimulation verändert. Dies ist im Versuchsaufbau vorgesehen. Bitte konzentrieren Sie sich ausschließlich auf den momentanen Schmerz und bewerten Sie ihn nur nach dem momentanen Gefühl.**
2. **Bitte vermeiden Sie unter allen Umständen Bewegungen des Kopfes. Bitte bewegen sie sich auch während der schmerzhaften Reizung nicht!**
3. **Den Unterschied zwischen der "Intensität" und dem "Unangenehmen" eines Reizes kann man sich am Beispiel eines Radios vorstellen:** Man kann eine Radiosendung unterschiedlich laut einstellen. Diese reine Lautstärke entspricht der

Schmerz*intensität* in unserem Versuch. Je lauter, desto intensiver. Wie *unangenehm* diese Lautstärke ist, wird auch dadurch bestimmt was gerade auf dem Sender ausgestrahlt wird. Ihr Lieblingslied mag intensiv Laut eingestellt, aber nicht unangenehm sein. Ein hohes, pfeifendes Testsignal mag dagegen schon bei geringer Lautstärke sehr unangenehm klingen. Kurz: Intensität entspricht Lautstärke, die unangenehme Seite entspricht dem Gefühl dabei.

4. **Für den ganzen Versuch gilt: Sollte Ihnen einmal ein Fehler unterlaufen ("Verdrücken"), dann drücken Sie bitte 5 Mal schnell hintereinander auf eine der Tasten, aber erst nach dem Sie beide Skalen abgeschlossen haben!** Dann kann der Versuchsleiter die Eintragung von der Auswertung ausschließen.
5. **Die Versuchsleiter können Ihre Bewertungen nicht direkt einsehen.**
6. **Sie erhalten einen Nottaster mit dem Sie den Versuch bei Bedarf jederzeit abbrechen können.**

Nach dem Versuch: Sicherstellen, dass die Stimmung des Probanden in Ordnung ist!
Haben sie Wünsche und Anregungen?
Gab es irgendwelche Besonderheiten die ihnen aufgefallen sind?

Supplement 4: Pictures selected from the International Affective Picture System (IAPS):

Selection criteria for IAPS pictures:

1. Valence rating (mean ratings of the mail standard population):
   - "Negative Set" (valence < 3.5)
   - "Neutral Set" (valence > 4.5 and valence < 5.5)
   - "Positive Set" (valence > 6.5)
2. Pictures depicting sharp objects, fire, phobic animals, and blood were excluded.
3. The Negative and Positive Sets were balanced with respect to arousal
4. The number of faces, humans, and animals in each set was balanced for the Negative, Positive and Neutral Sets [2]
5. All four sets were balanced for mean picture luminance.

"**Positive Set**" (mean valence = 7.3 ± 0.4, mean arousal = 5.08 ± 1.18)
IAPS-Reference-Numbers: 1440, 1441, 1460, 1463, 1710, 1750, 2035, 2058, 2071, 2340, 2341, 2660, 4001, 4141, 4142, 4225, 4235, 4255, 4599, 4601, 5000, 5200, 5220, 5270, 5300, 5594, 5600, 5700, 5780, 5825, 7260, 7405, 8186, 8380, 8461, 8497

"**Neutral Set**" (mean valence = 5.0 ± 0.3, mean arousal = 3.5 ± 0.8)
IAPS-Reference-Numbers: 1390, 1505, 1617, 1945, 2020, 2038, 2102, 2200, 2214, 2270, 2280, 2390, 2400, 2441, 2512, 4000, 4100, 4503, 4525, 4542, 5510, 5531, 5533, 5740, 6150, 7000, 7001, 7002, 7003, 7020, 7032, 7035, 7255, 7287, 7506, 9070

"**Negative Set**" (mean valence = 2.6 ± 0.4, mean arousal = 5.2 ± 0.8)
IAPS-Reference-Numbers: 2095, 2141, 2205, 2375.1, 2683, 2703, 2799, 3005.1, 3350, 3500, 6212, 6230, 6260, 9000, 9001, 9040, 9075, 9140, 9184, 9185, 9220, 9280, 9290, 9295, 9302, 9322, 9340, 9413, 9419, 9425, 9520, 9560, 9901, 9904, 9911, 9920

"Scrambled Set"
Twelve pictures of each set were used to create the "Scrambled Set", which consisted of 36 pictures. A Fourier type scrambling method at 0.01 % phase coherence [6] was used. The scrambled pictures matched the original pictures in luminance and mean color composition while showing no objective content.

An additional, different set of pictures was used for the training session:

“**Positive Pre-Testing Set**”
IAPS-Reference-Numbers: 1540, 1722, 1811, 2045, 2274, 2347, 2550, 4002, 4090, 4240, 5623, 5660, 5811, 7480 ,7492 ,8185 ,8200, 8503
“**Neutral Pre-Testing Set**”
IAPS-Reference-Numbers: 1645, 1908, 2005, 2191, 2210, 2272, 2385, 2396, 4500, 4533, 5120, 5500, 5731, 7004, 7006, 7017 ,7235, 7497
“**Negative Pre-Testing Set**”
IAPS-Reference-Numbers: 2700, 2750, 2800, 6263, 6563, 6838, 9010, 9041, 9090, 9163, 9182, 9291, 9301, 9342, 9430, 9571, 9600, 9908

Supplement 5: Multi-voxel pattern training and cross-validation

We roughly followed the strategy used by Wager and colleagues [12], with a few notable differences:

- We aimed at training weights for our specific sample (i.e. healthy, young males having received a nasal spray), not for the general population.
- Instead of using raw data for training, we used the beta-maps obtained from first level analysis with SPM8. Using beta-maps generated by SPM's general linear model allows for controlling confounds, such as movement artifacts and signal drifts before predictor training.
- Our training-sample for MVPA analysis contained more participants, a finer resolution of the temperature range and was obtained at higher field strength (3 Tesla, instead of 1.5 Tesla). This allowed us to perform machine learning on whole brain data without the need for feature pre-selection (ROIs determined by reverse inference).

We trained a multi-voxel weights-map able to predict applied physical temperature intensity from the whole-brain activity patterns obtained during heat perception. We used the first-level beta-maps obtained for the parallel study [13] for this purpose. Images from both studies were pre-processed identically. Both the placebo and the oxytocin session from the original study [13] were included to avoid biasing predictions towards one medication or the other. Further, we defined temperature, rather than VAS-intensity or -unpleasantness ratings as prediction target, since there were small, but significant medication effects on ratings in the training set, which could bias predictions [13].

For each subject and session, eight first-level whole-brain beta maps representing the eight heat temperature conditions were used as predictive features. The beta maps were standardized by-voxel (z-scores) and underwent dimensionality reduction using principal

component analysis (PCA). PCA allowed condensing the number of candidate features from 318677 voxel to 469 principal components, while retaining 99.9% of variance (see: Fig. S4.1).

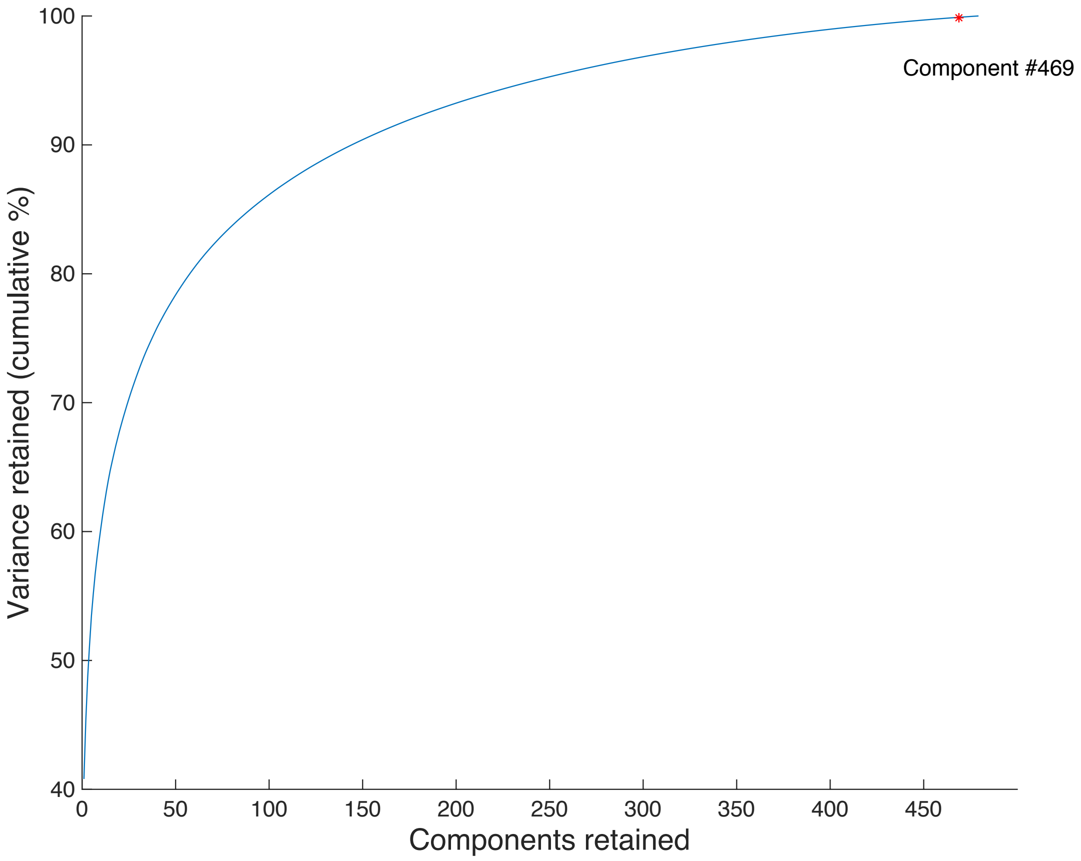

Fig. S4.1: Dimensionality reduction of training data: Number of principal components selected versus variance retained: 469 out of 479 components were required in order to retain 99.9% variance.

A least-squares linear regression algorithm with elastic-net regularization (*lasso* function, Statistics and Machine Learning Toolbox, MATLAB 2014b) was used for parameter estimation [10]. Leave-one-subject-out cross-validation (LOSO-CV) was performed with custom code in order to estimate the generalizability of results to new subjects from our target population.

The regularization parameter *lambda* was adjusted in order to optimize cross-validation error. This increases generalizability of the results at the cost of introducing a prediction bias ("bias-variance trade-off", Tibshirani, 1996). After scanning a range of *lambda* values

between 10^{-7} and 10^{7}, a value of 32 was found to minimize cross-validation error (see: Fig. S4.2).

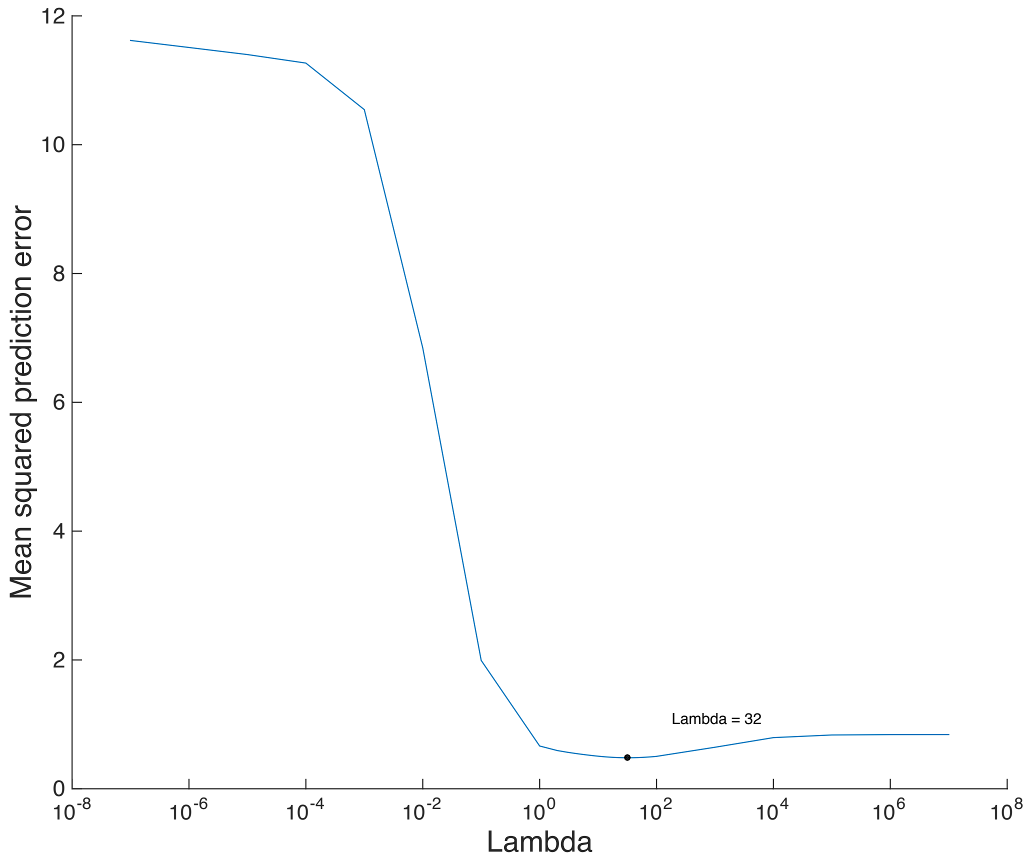

Fig. S4.2: Values of the regularization parameter lambda, plotted against the overall prediction error as obtained by leave-one-session-out cross-validation.

After fixing the lambda value to 32, the elastic-net parameter *alpha* was gradually increased in order to improve the sparsity of the parameter-matrix. An *alpha* of 0.0019 was determined as optimal in terms of mean squared cross-validation error (Fig. S4.3). With this procedure 205 out of 469 parameter estimates contributing little to the predictive performance were fixed at zero. This effectively eliminated the contributions from the corresponding principal components to the final mask.

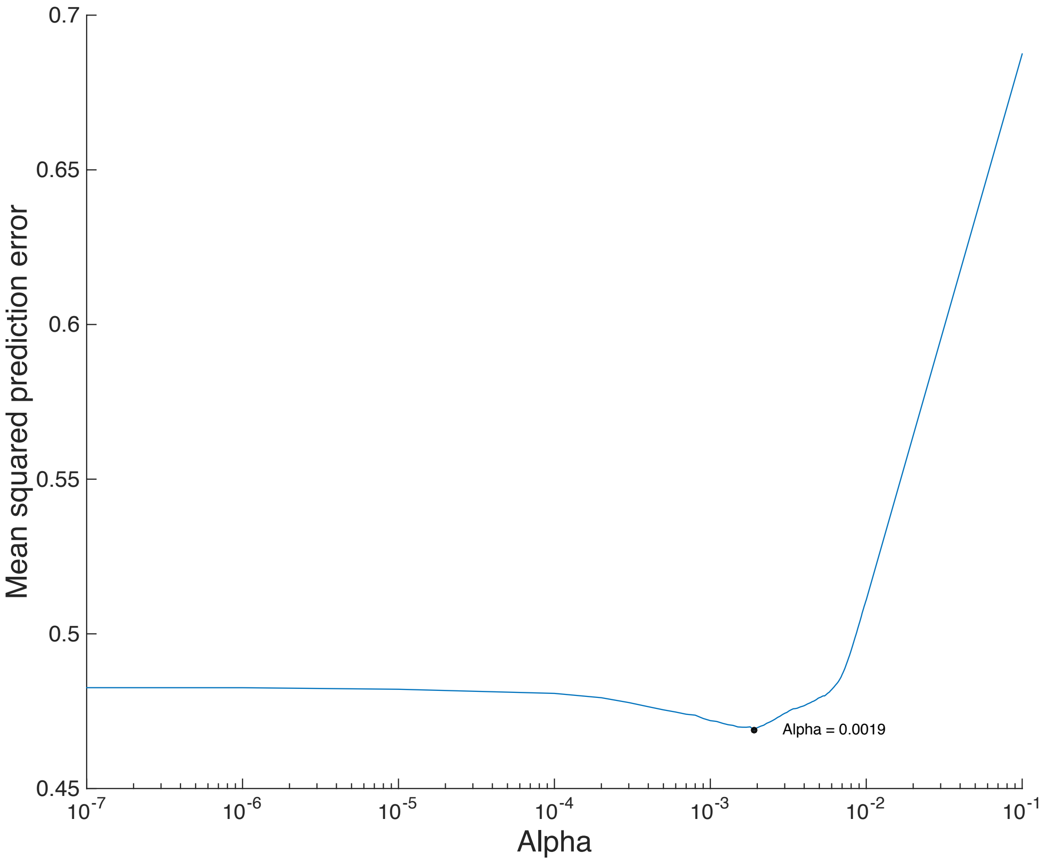

Fig. S4.3: Values of the shrinkage parameter alpha, plotted against the overall prediction error as obtained by leave-one-session-out cross-validation.

After lambda and alpha selection, the correspondence between predicted and actual temperatures was satisfactorily (r=0.67), with a mean prediction error of 0.68°C (see: Fig. S4.4). The correlation between temperatures predicted from brain images and heat intensity ratings was equally high (r=0.67, see: Fig. S4.5). This indicates that predictions made from fMRI images corresponded reliably to both applied thermode temperature and perceived heat intensity. As expected, the regularization procedure introduced a conservative bias: Lower temperatures were over- and higher temperatures under-estimated (see: Fig. S4.4). We therefore refrain from using the unit °C for the temperature predictions used from here on.

In conclusion, CV indicated that our optimized weights were reasonably predictive for novel data from the sample population, with a conservative bias towards predicting temperatures to be more similar than actual. Of note, adjusting lambda and alpha based on CV-estimation

error will bias the estimated generalization error towards more optimistic results [11]. Therefore, the predictive performance of predictor weights on the test-data (see: Results section of the main manuscript) should provide a more accurate estimate of generalization error.

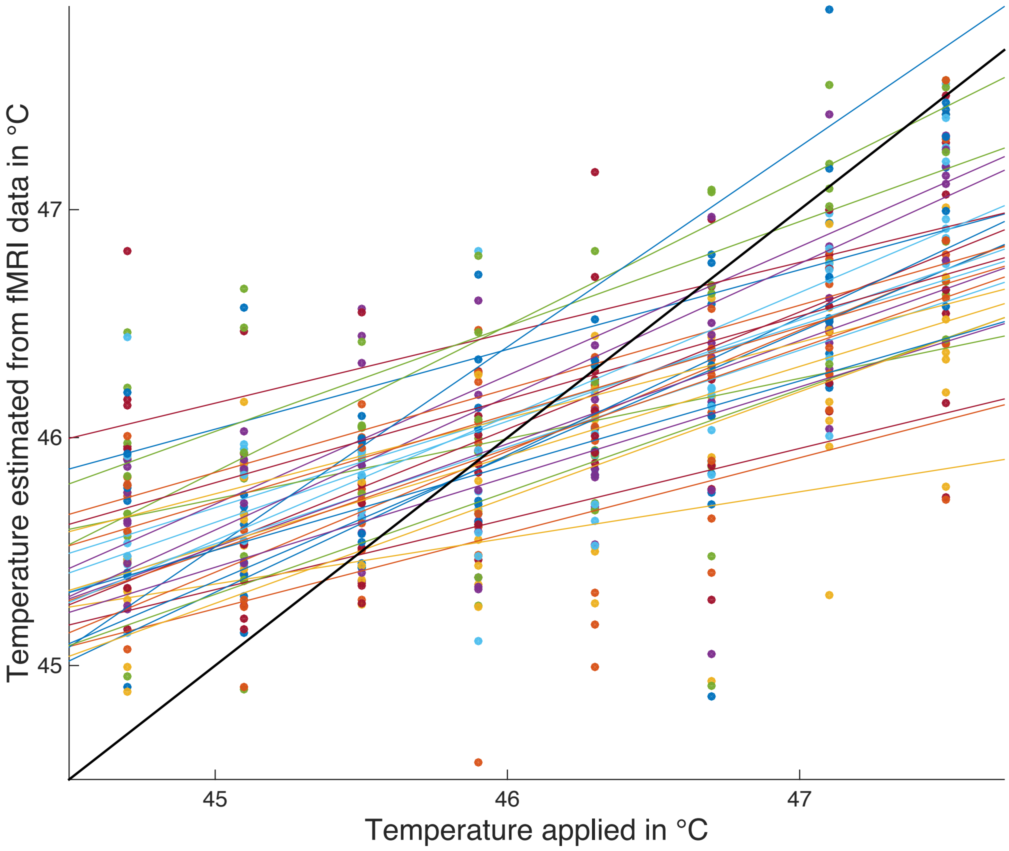

Fig. S4.4: Applied temperatures plotted against temperatures predicted from the participants iteratively held out of the training procedure (leave-one-subject out cross-validation). Best-fitting linear regressions for each subject are shown by the correspondingly colored lines. Estimates were obtained after a shrinkage (lambda = 32, alpha = 0.0019), which eliminated 205 of the 469 parameters. Ordinary least-squares interpolation lines were added for each participant. Different colors denote different participants. A diagonal reference line (black) was added to illustrate the systematic deviation from unbiased fit.

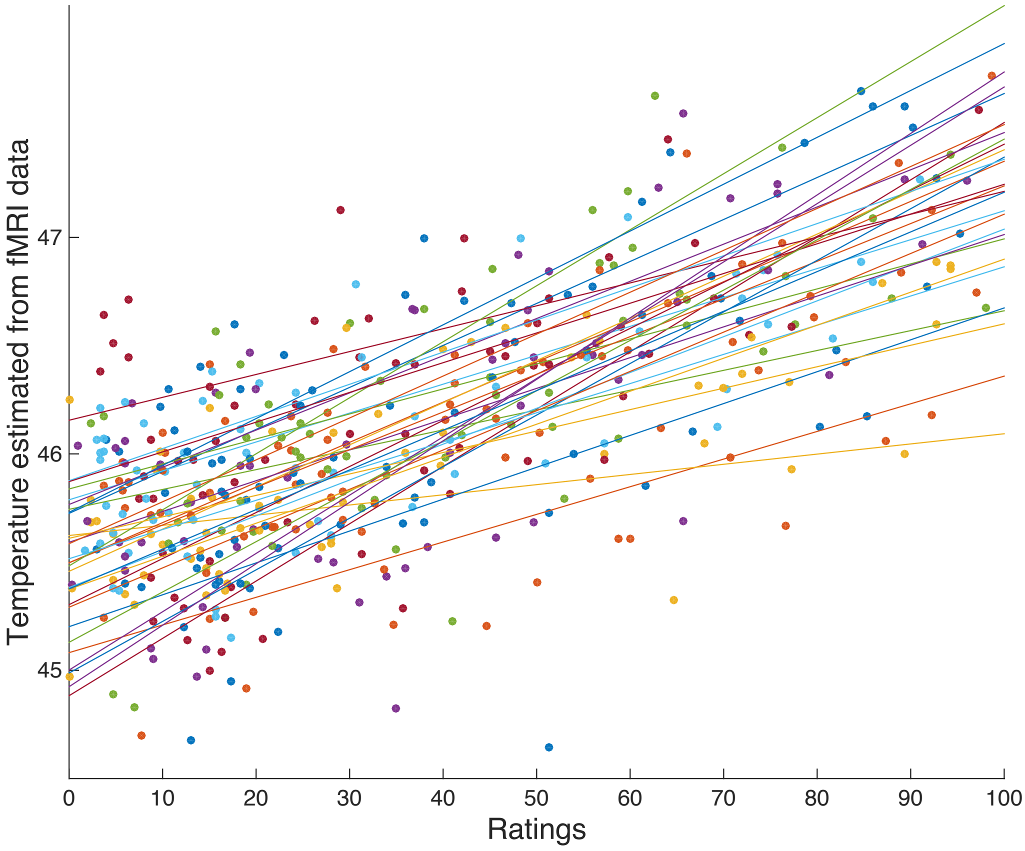

Fig. S4.5: Heat intensity ratings plotted against temperatures predicted from participants iteratively held out of the training procedure (leave-one-subject out cross-validation). Estimates were obtained after a shrinkage (lambda = 32, alpha = 0.0019), which eliminated 205 of the 469 parameters. Ordinary least-squares interpolation lines were added for each participant. Different colors denote different participants.

As a last step, the weights-map was obtained by performing training with data from all participants and optimized lambda and alpha. The obtained weights were projected back to voxel space by using the original PCA-coefficients.

Further Diagnostics

In order to identify the main sources of prediction error, training and cross-validation of the shrinkage-optimized model was repeated with varying training-set sizes. Fig. S4.6 depicts the resulting "learning curve", indicating that the cross-validation error outweighed training error (bias) at the given training set size. The major source of prediction error for the present

machine-learning problem may therefore be high between-subject and image variance, rather than a biased prediction model (underfitting). Nevertheless, higher-order polynomial models, in combination with larger sample sizes, may allow for more precise and unbiased predictions of perceived heat pain stimulus intensity from whole-brain neuroimaging-data in the future.

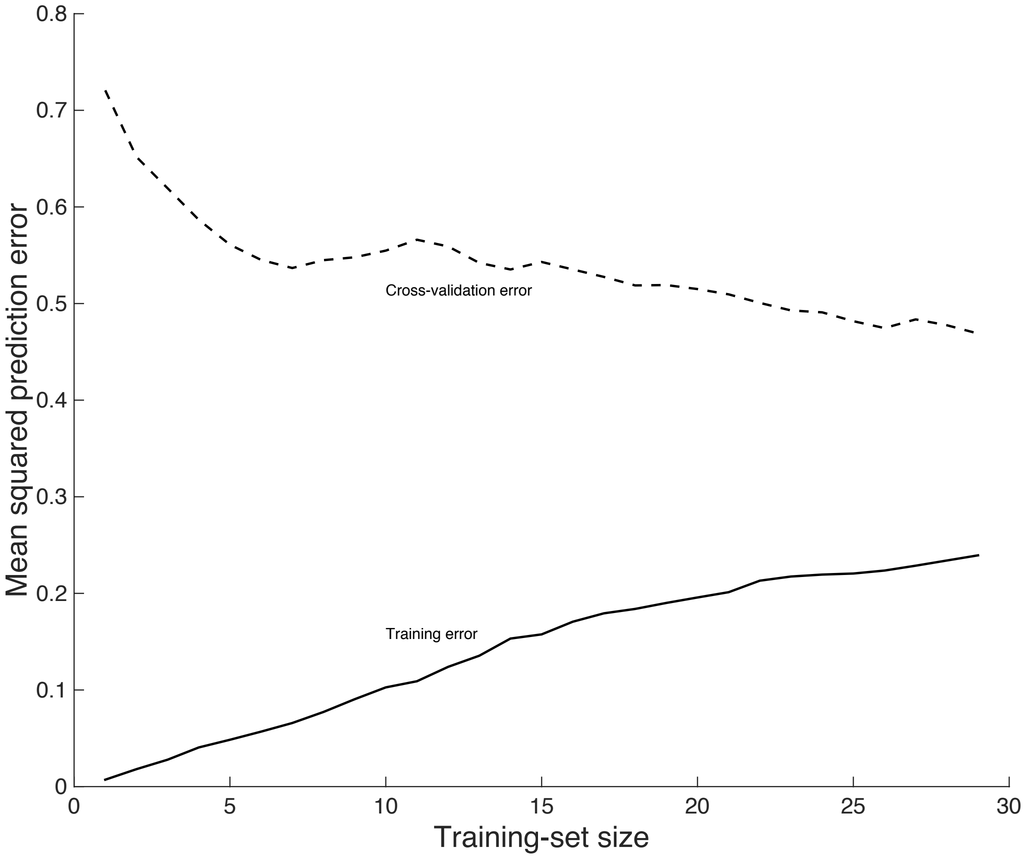

Fig. S4.6: Training and cross-validation error, estimated at varying training-set size.

Supplement 6: Analysis syntax for R and complete results output

```
#### Set up environment/dataset #####
#### CLEAR WORKSPACE
rm(list = ls())
#### LOAD LIBRARIES
library(lme4)
library(lmerTest) # will enhance the lme4 summary by df's and p-values
library(pbkrtest) # required by lmerTest for Kenward-Roger's
library(ggplot2)
library(GGally)
library(grid)
library(plyr)
library(lsmeans)
library(gridExtra)
library(Rmisc)
library(psychometric)
#### Set basic graphing features
respx=1200 # resolution in dots/inch
gwidth=80 # width in mm  for single plots
gheight=gwidth/2 # heigth in mm  for single plots
gwidthtotal=80 # width in mm  for multiplots
#### LOAD DATA
load("Oxy2.Rda")
#### Assign Factor Lables
Oxy2$Medication<- factor(Oxy2$Medication,
                    labels = c("Placebo", "Oxytocin"))
Oxy2$Temperature<- factor(Oxy2$Temperature,
                     levels = c(35,44.7,47.1),
                     labels = c("35.0°C", "44.7°C", "47.1°C"))

#### ANALYSIS GENERAL: ####
#### Rating Duration
CI(Oxy2$RatingDuration, ci = 0.95)
#### Group Allocation
group=aggregate(Oxy2$OxyFirst,by=list(Oxy2$Proband),FUN=mean,na.rm=TRUE)
mean(group$x)
#### Select data for main aims (excluding 35°C and Scrambled) and z-Transform for a better mixed-model performance
Oxywo35woScr<-subset(Oxy2, Temperature != "35.0°C" & Emo != 'scrambled')
Oxywo35woScr$Emo<-factor(Oxywo35woScr$Emo) #Reassign factors to remove factor level scrambled
Oxywo35woScr$Temperature<-factor(Oxywo35woScr$Temperature) #Reassign factors to remove factor level 35
Oxywo35woScr$ZIntensity=as.numeric(scale(Oxywo35woScr$Intensity, center = TRUE, scale = TRUE))
Oxywo35woScr$ZUnpleasantness=as.numeric(scale(Oxywo35woScr$Unpleasantness, center = TRUE, scale = TRUE))
Oxywo35woScr$ZMVPA=as.numeric(scale(Oxywo35woScr$MVPA, center = TRUE, scale = TRUE))

#### ANALYSIS INTENSITY MAIN ####
```

```
# INTENSITY (Standardized)
convergenceScaling=1 # Scaling factor necessary for some variables to achieve convergence in gradient descent
# Calculates Model
MM_int_full<-lmer(ZIntensity*convergenceScaling~(Medication+Temperature+Emo)^3+
        (1+(Medication+Temperature+Emo)^2|Proband),
       data=Oxywo35woScr,REML=TRUE, control=lmerControl(optCtrl=list(maxfun=180000)),na.action =na.omit)
# Generates Summary Output for Model
summary(MM_int_full)
# Generates ANOVA Statistic via lmerTest
anova(MM_int_full,ddf = "Kenward-Roger")

#### Estimate Marginal Means using lsmeans by Russell V. Lenth The University of Iowa October 9, 2015
gridInt=ref.grid(MM_int_full) # Create the reference grid for all comparisons
#Estimate Marginal Means for TEMPERATURE
EMMintTemp<- lsmeans(gridInt,~Temperature)
CInt.Temp<-pairs(EMMintTemp) #Test pairwise differences
CInt.Temp.Results<-show(CInt.Temp)
#Standardized Effect
CInt.Temp.ZResults<-CInt.Temp.Results
CInt.Temp.ZResults$estimate<-CInt.Temp.ZResults$estimate/convergenceScaling # Re-Scale contrast estimates to SD
CInt.Temp.ZResults$SE<-CInt.Temp.ZResults$SE/convergenceScaling
CInt.Temp.ZResults$CIlo<-CInt.Temp.ZResults$estimate-(CInt.Temp.ZResults$SE*qt(.975, df=CInt.Temp.ZResults$df))
CInt.Temp.ZResults$CIhi<-CInt.Temp.ZResults$estimate+(CInt.Temp.ZResults$SE*qt(.975, df=CInt.Temp.ZResults$df))
CInt.Temp.ZResults
#Non-Standardized Effect
CInt.Temp.Results$estimate<-
CInt.Temp.Results$estimate*sd(Oxywo35woScr$Intensity,na.rm=TRUE)/convergenceScaling # Re-Scale contrast estimates
and CI's from z-Scores
CInt.Temp.Results$SE<-CInt.Temp.Results$SE*sd(Oxywo35woScr$Intensity,na.rm=TRUE)/convergenceScaling
CInt.Temp.Results$CIlo<-CInt.Temp.Results$estimate-(CInt.Temp.Results$SE*qt(.975, df=CInt.Temp.Results$df))
CInt.Temp.Results$CIhi<-CInt.Temp.Results$estimate+(CInt.Temp.Results$SE*qt(.975, df=CInt.Temp.Results$df))
CInt.Temp.Results
#Estimate Marginal Means for EMOTION
EMMintEmo<- lsmeans(gridInt,~Emo)
CInt.Emo<-pairs(EMMintEmo) #Test pairwise differences.
CInt.Emo.Results<-show(CInt.Emo)
#Standardized Effect
CInt.Emo.ZResults<-CInt.Emo.Results
CInt.Emo.ZResults$estimate<-CInt.Emo.ZResults$estimate/convergenceScaling # Re-Scale contrast estimates to SD
CInt.Emo.ZResults$SE<-CInt.Emo.ZResults$SE/convergenceScaling
CInt.Emo.ZResults$CIlo<-CInt.Emo.ZResults$estimate-(CInt.Emo.ZResults$SE*qt(.975, df=CInt.Emo.ZResults$df))
CInt.Emo.ZResults$CIhi<-CInt.Emo.ZResults$estimate+(CInt.Emo.ZResults$SE*qt(.975, df=CInt.Emo.ZResults$df))
CInt.Emo.ZResults
#Non-Standardized Effect
CInt.Emo.Results$estimate<-CInt.Emo.Results$estimate*sd(Oxywo35woScr$Intensity,na.rm=TRUE)/convergenceScaling
# Re-Scale contrast estimates and CI's from z-Scores to raw Scaling
```

```
CInt.Emo.Results$SE<-CInt.Emo.Results$SE*sd(Oxywo35woScr$Intensity,na.rm=TRUE)/convergenceScaling
CInt.Emo.Results$CIlo<-CInt.Emo.Results$estimate-(CInt.Emo.Results$SE*qt(.975, df=CInt.Emo.Results$df))
CInt.Emo.Results$CIhi<-CInt.Emo.Results$estimate+(CInt.Emo.Results$SE*qt(.975, df=CInt.Emo.Results$df))
CInt.Emo.Results
#Estimate Marginal Means for MEDICATION:TEMPERATURE
EMMinttemp<- lsmeans(gridInt,~Medication:Temperature)
CInt.MedTemp<-pairs(EMMinttemp)
CInt.MedTemp.Results<-show(CInt.MedTemp)
#Standardized Effect
CInt.MedTemp.ZResults<-CInt.MedTemp.Results
CInt.MedTemp.ZResults$estimate<-CInt.MedTemp.ZResults$estimate/convergenceScaling # Re-Scale contrast estimates to
SD
CInt.MedTemp.ZResults$SE<-CInt.MedTemp.ZResults$SE/convergenceScaling
CInt.MedTemp.ZResults$CIlo<-CInt.MedTemp.ZResults$estimate-(CInt.MedTemp.ZResults$SE*qt(.975,
df=CInt.MedTemp.ZResults$df))
CInt.MedTemp.ZResults$CIhi<-CInt.MedTemp.ZResults$estimate+(CInt.MedTemp.ZResults$SE*qt(.975,
df=CInt.MedTemp.ZResults$df))
CInt.MedTemp.ZResults
#Non-Standardized Effect
CInt.MedTemp.Results$estimate<-
CInt.MedTemp.Results$estimate*sd(Oxywo35woScr$Intensity,na.rm=TRUE)/convergenceScaling # Re-Scale contrast
estimates and CI's from z-Scores to raw Scaling
CInt.MedTemp.Results$SE<-CInt.MedTemp.Results$SE*sd(Oxywo35woScr$Intensity,na.rm=TRUE)/convergenceScaling
CInt.MedTemp.Results$CIlo<-CInt.MedTemp.Results$estimate-(CInt.MedTemp.Results$SE*qt(.975,
df=CInt.MedTemp.Results$df))
CInt.MedTemp.Results$CIhi<-CInt.MedTemp.Results$estimate+(CInt.MedTemp.Results$SE*qt(.975,
df=CInt.MedTemp.Results$df))
CInt.MedTemp.Results
# Means for graphing the interaction effect
EMMintprinttemp<-show(EMMinttemp)
EMMintprinttemp$lsmean<-
EMMintprinttemp$lsmean*sd(Oxywo35woScr$Intensity,na.rm=TRUE)/convergenceScaling+mean(Oxywo35woScr$Intens
ity,na.rm=TRUE) #Transform EMM back to raw scale (inverse z-Transform)
EMMintprinttemp$SE<-EMMintprinttemp$SE*sd(Oxywo35woScr$Intensity,na.rm=TRUE)/convergenceScaling
EMMintprinttemp$lower.CL<-
EMMintprinttemp$lower.CL*sd(Oxywo35woScr$Intensity,na.rm=TRUE)/convergenceScaling+mean(Oxywo35woScr$Inte
nsity,na.rm=TRUE)
EMMintprinttemp$upper.CL<-
EMMintprinttemp$upper.CL*sd(Oxywo35woScr$Intensity,na.rm=TRUE)/convergenceScaling+mean(Oxywo35woScr$Inte
nsity,na.rm=TRUE)
# Estimate Marginal Means for Plot
EMMint<- lsmeans(gridInt,~Medication:Temperature:Emo)
EMMintprint<-show(EMMint)
# Backtransform standardized estimates to actual ratings
```

```
EMMintprint$lsmean<-
EMMintprint$lsmean*sd(Oxywo35woScr$Intensity,na.rm=TRUE)/convergenceScaling+mean(Oxywo35woScr$Intensity,n
a.rm=TRUE) #Transform EMM back to raw scale (inverse z-Transform)
EMMintprint$SE<-EMMintprint$SE*sd(Oxywo35woScr$Intensity,na.rm=TRUE)/convergenceScaling
EMMintprint$lower.CL<-
EMMintprint$lower.CL*sd(Oxywo35woScr$Intensity,na.rm=TRUE)/convergenceScaling+mean(Oxywo35woScr$Intensity
,na.rm=TRUE)
EMMintprint$upper.CL<-
EMMintprint$upper.CL*sd(Oxywo35woScr$Intensity,na.rm=TRUE)/convergenceScaling+mean(Oxywo35woScr$Intensity
,na.rm=TRUE)

#### Plot Intensity
adddist=30
infomax=60
intplot=ggplot() +
  geom_jitter(data=Oxywo35woScr,      aes(x = Medication, y = Intensity, group=Emo, color=Emo,fill = Emo),
position=position_jitterdodge(jitter.width = 0.2,dodge.width=0.7),alpha=0.2) +   #Single-Value Dots
  #Annotation for Medication*Temp as horizontal lines + text
    geom_line(data = subset(EMMintprinttemp,  Temperature == '44.7°C' & Medication == 'Placebo'),
            aes( y = lsmean+adddist, x=c(0.7,1.3)),color="#999999")+
    geom_line(data = subset(EMMintprinttemp,  Temperature == '44.7°C' & Medication == 'Placebo'),
            aes( y = c(lsmean+adddist,infomax), x=1),color="#999999")+
    geom_line(data = subset(EMMintprinttemp,  Temperature == '44.7°C' & Medication == 'Oxytocin'),
             aes( y = lsmean+adddist, x=c(1.7,2.3)),color="#999999")+
    geom_line(data = subset(EMMintprinttemp,  Temperature == '44.7°C' & Medication == 'Oxytocin'),
              aes( y = c(lsmean+adddist,infomax), x=2),color="#999999")+
    geom_line(data = subset(EMMintprinttemp,  Temperature == '44.7°C' & Medication == 'Placebo'),
            aes( y = c(infomax,infomax), x=c(1,2)),color="#999999")+
    geom_text(data = subset(EMMintprinttemp,  Temperature == '44.7°C' & Medication == 'Placebo'),
            aes( y = infomax+5, x=1.5, label='*'), size = 8,color="#999999")+
  guides(fill=FALSE)+
  facet_grid( ~ Temperature)+   #Split grid by Temperature
#Adds Means and Error-Bars for each Condition
  geom_point(data=EMMintprint,    aes(x = Medication, y = lsmean, group=Emo, color=Emo),
position=position_dodge(width=0.7),  size = 3)+
  geom_errorbar(data=EMMintprint, aes(x = Medication, y = lsmean, group=Emo, color=Emo, ymax = upper.CL, ymin =
lower.CL),position=position_dodge(width=0.7),width=0,  size = 1.5)+
  scale_colour_manual("Emotion:",values = c("#0072B2", "#666666", "#CC79A7"),labels= c("negative", "neutral",
"positive"))+   #Change Legend Nameing and Position
  scale_y_continuous("Heat intensity rating")+ #Change axis legend
  theme(legend.position='bottom',legend.direction
="horizontal",legend.text=element_text(size=12),legend.title=element_text(size=14,face="bold"),legend.key=element_blank
())+
  ggtitle("a)")+theme(plot.title = element_text(hjust = 0))+
  theme(aspect.ratio = 1/sqrt(2))+ #fix plot size
  theme(axis.text=element_text(size=12), #fix axis font size
```

```
      axis.title=element_text(size=14,face="bold"))+ #fix axis title font size
  theme(strip.text.x = element_text(size = 12,face="bold"))+
  theme(panel.grid.major.y = element_line(colour = "#111111",size=0.2), #fix grid display
      panel.grid.minor.y = element_blank(), #fix grid display
      panel.grid.major.x = element_blank(),
      axis.title.x= element_blank(),
      panel.grid.minor = element_blank(),
      strip.background = element_rect(fill = 'transparent'),
      panel.background = element_blank(),
      axis.line = element_line(colour = "black"))
intplot
ggsave(
  "Figure3a.pdf",
  plot = intplot,
  unit=c("mm"),
  width = gwidth,
  height = gheight,
  scale=3
)

#### ANALYSIS UNPLEASANTNESS MAIN ####
#  UNPLEASANTNESS (Standardized)
convergenceScaling=1 # Scaling factor necessary for some variables to achieve convergence in gradient descent
# Calculate Model
MM_unp_full<-lmer(ZUnpleasantness*convergenceScaling~(Medication+Temperature+Emo)^3+
             (1+(Medication+Temperature+Emo)^2|Proband),
            data=Oxywo35woScr,REML=TRUE, control=lmerControl(optCtrl=list(maxfun=180000)),na.action =na.omit)
# Generates Summary Output for Model
summary(MM_unp_full)
# Generates ANOVA Statistiv via lmerTest
anova(MM_unp_full,ddf = "Kenward-Roger")

# Estimate Marginal Means using lsmeans by Russell V. Lenth The University of Iowa October 9, 2015
gridunp=ref.grid(MM_unp_full) # Create the reference grid for all comparisons
#Estimate Marginal Means for TEMPERATURE
EMMunpTemp<- lsmeans(gridunp,~Temperature)
Cunp.Temp<-pairs(EMMunpTemp) #Test pairwise differences
Cunp.Temp.Results<-show(Cunp.Temp)
#Standardized Effect
Cunp.Temp.ZResults<-Cunp.Temp.Results
Cunp.Temp.ZResults$estimate<-Cunp.Temp.ZResults$estimate/convergenceScaling # Re-Scale contrast estimates to SD
Cunp.Temp.ZResults$SE<-Cunp.Temp.ZResults$SE/convergenceScaling
Cunp.Temp.ZResults$CIlo<-Cunp.Temp.ZResults$estimate-(Cunp.Temp.ZResults$SE*qt(.975,
df=Cunp.Temp.ZResults$df))
Cunp.Temp.ZResults$CIhi<-Cunp.Temp.ZResults$estimate+(Cunp.Temp.ZResults$SE*qt(.975,
df=Cunp.Temp.ZResults$df))
```

Cunp.Temp.ZResults
#Non-Standardized Effect
Cunp.Temp.Results$estimate<-
Cunp.Temp.Results$estimate*sd(Oxywo35woScr$Unpleasantness,na.rm=TRUE)/convergenceScaling # Re-Scale contrast estimates and CI's from z-Scores to raw Scaling
Cunp.Temp.Results$SE<-Cunp.Temp.Results$SE*sd(Oxywo35woScr$Unpleasantness,na.rm=TRUE)/convergenceScaling
Cunp.Temp.Results$CIlo<-Cunp.Temp.Results$estimate-(Cunp.Temp.Results$SE*qt(.975, df=Cunp.Temp.Results$df))
Cunp.Temp.Results$CIhi<-Cunp.Temp.Results$estimate+(Cunp.Temp.Results$SE*qt(.975, df=Cunp.Temp.Results$df))
Cunp.Temp.Results
#Estimate Marginal Means for EMOTION
EMMunpEmo<- lsmeans(gridunp,~Emo)
Cunp.Emo<-pairs(EMMunpEmo) #Test pairwise differences.
Cunp.Emo.Results<-show(Cunp.Emo)
#Standardized Effect
Cunp.Emo.ZResults<-Cunp.Emo.Results
Cunp.Emo.ZResults$estimate<-Cunp.Emo.ZResults$estimate/convergenceScaling # Re-Scale contrast estimates to SD
Cunp.Emo.ZResults$SE<-Cunp.Emo.ZResults$SE/convergenceScaling
Cunp.Emo.ZResults$CIlo<-Cunp.Emo.ZResults$estimate-(Cunp.Emo.ZResults$SE*qt(.975, df=Cunp.Emo.ZResults$df))
Cunp.Emo.ZResults$CIhi<-Cunp.Emo.ZResults$estimate+(Cunp.Emo.ZResults$SE*qt(.975, df=Cunp.Emo.ZResults$df))
Cunp.Emo.ZResults
#Non-Standardized Effect
Cunp.Emo.Results$estimate<-
Cunp.Emo.Results$estimate*sd(Oxywo35woScr$Unpleasantness,na.rm=TRUE)/convergenceScaling # Re-Scale contrast estimates and CI's from z-Scores to raw Scaling
Cunp.Emo.Results$SE<-Cunp.Emo.Results$SE*sd(Oxywo35woScr$Unpleasantness,na.rm=TRUE)/convergenceScaling
Cunp.Emo.Results$CIlo<-Cunp.Emo.Results$estimate-(Cunp.Emo.Results$SE*qt(.975, df=Cunp.Emo.Results$df))
Cunp.Emo.Results$CIhi<-Cunp.Emo.Results$estimate+(Cunp.Emo.Results$SE*qt(.975, df=Cunp.Emo.Results$df))
Cunp.Emo.Results
#Estimate Marginal Means for MEDICATION:TEMPERATURE
EMMunptemp<- lsmeans(gridunp,~Medication:Temperature)
Cunp.MedTemp<-pairs(EMMunptemp)
Cunp.MedTemp.Results<-show(Cunp.MedTemp)
#Standardized Effect
Cunp.MedTemp.ZResults<-Cunp.MedTemp.Results
Cunp.MedTemp.ZResults$estimate<-Cunp.MedTemp.ZResults$estimate/convergenceScaling # Re-Scale contrast estimates to SD
Cunp.MedTemp.ZResults$SE<-Cunp.MedTemp.ZResults$SE/convergenceScaling
Cunp.MedTemp.ZResults$CIlo<-Cunp.MedTemp.ZResults$estimate-(Cunp.MedTemp.ZResults$SE*qt(.975, df=Cunp.MedTemp.ZResults$df))
Cunp.MedTemp.ZResults$CIhi<-Cunp.MedTemp.ZResults$estimate+(Cunp.MedTemp.ZResults$SE*qt(.975, df=Cunp.MedTemp.ZResults$df))
Cunp.MedTemp.ZResults
#Non-Standardized Effect
Cunp.MedTemp.Results$estimate<-
Cunp.MedTemp.Results$estimate*sd(Oxywo35woScr$Unpleasantness,na.rm=TRUE)/convergenceScaling # Re-Scale contrast estimates and CI's from z-Scores to raw Scaling

```
Cunp.MedTemp.Results$SE<-
Cunp.MedTemp.Results$SE*sd(Oxywo35woScr$Unpleasantness,na.rm=TRUE)/convergenceScaling
Cunp.MedTemp.Results$CIlo<-Cunp.MedTemp.Results$estimate-(Cunp.MedTemp.Results$SE*qt(.975,
df=Cunp.MedTemp.Results$df))
Cunp.MedTemp.Results$CIhi<-Cunp.MedTemp.Results$estimate+(Cunp.MedTemp.Results$SE*qt(.975,
df=Cunp.MedTemp.Results$df))
Cunp.MedTemp.Results
#Estimate Marginal Means for MEDICATION:TEMPERATURE
EMMunp<- lsmeans(gridunp,~Medication:Temperature:Emo)
Cunp.3way<-pairs(EMMunp)
Cunp.3way.results<-show(Cunp.3way)
Cunp.3way.Zresults<-Cunp.3way.results
#Standardized Effect
Cunp.3way.Zresults$estimate<-Cunp.3way.Zresults$estimate/convergenceScaling
Cunp.3way.Zresults$SE<-Cunp.3way.Zresults$SE/convergenceScaling
Cunp.3way.Zresults$CIlo<-Cunp.3way.Zresults$estimate-(Cunp.3way.Zresults$SE*qt(.975, df=Cunp.3way.Zresults$df))
Cunp.3way.Zresults$CIhi<-Cunp.3way.Zresults$estimate+(Cunp.3way.Zresults$SE*qt(.975, df=Cunp.3way.Zresults$df))
Cunp.3way.Zresults
#Non-Standardized Effect
Cunp.3way.results$estimate<-
Cunp.3way.results$estimate*sd(Oxywo35woScr$Unpleasantness,na.rm=TRUE)/convergenceScaling
Cunp.3way.results$SE<-Cunp.3way.results$SE*sd(Oxywo35woScr$Unpleasantness,na.rm=TRUE)/convergenceScaling
Cunp.3way.results$CIlo<-Cunp.3way.results$estimate-(Cunp.3way.results$SE*qt(.975, df=Cunp.3way.results$df))
Cunp.3way.results$CIhi<-Cunp.3way.results$estimate+(Cunp.3way.results$SE*qt(.975, df=Cunp.3way.results$df))
Cunp.3way.results
# Estimate Marginal Means for Plot
EMMunpprint<-show(EMMunp)
# Backtransform standardized estimates to actual ratings
EMMunpprint$lsmean<-
EMMunpprint$lsmean*sd(Oxywo35woScr$Unpleasantness,na.rm=TRUE)/convergenceScaling+mean(Oxywo35woScr$Un
pleasantness,na.rm=TRUE) #Transform EMM back to raw scale (inverse z-Transform)
EMMunpprint$SE<-EMMunpprint$SE*sd(Oxywo35woScr$Unpleasantness,na.rm=TRUE)/convergenceScaling
EMMunpprint$lower.CL<-
EMMunpprint$lower.CL*sd(Oxywo35woScr$Unpleasantness,na.rm=TRUE)/convergenceScaling+mean(Oxywo35woScr$
Unpleasantness,na.rm=TRUE)
EMMunpprint$upper.CL<-
EMMunpprint$upper.CL*sd(Oxywo35woScr$Unpleasantness,na.rm=TRUE)/convergenceScaling+mean(Oxywo35woScr$
Unpleasantness,na.rm=TRUE)

#Plot Unpleasantness
adddist=-10
infomax=20
unpplot=ggplot() +
  geom_jitter(data=Oxywo35woScr,       aes(x = Medication, y = Unpleasantness, group=Emo, color=Emo,fill = Emo),
position=position_jitterdodge(jitter.width = 0.2,dodge.width=0.7),alpha=0.2) +   #Single-Value Dots
  # Annotation for 3-Way interaction effect
```

```
  geom_line(data = subset(EMMunpprint,  Temperature == '47.1°C' & Medication == 'Oxytocin' & Emo=='negative'),
          aes( y = c(lsmean+adddist,infomax), x=1.765),color="#999999")+
  geom_line(data = subset(EMMunpprint,  Temperature == '47.1°C' & Medication == 'Oxytocin' & Emo=='positive'),
          aes( y = c(lsmean+adddist,infomax), x=2.235),color="#999999")+
  geom_line(data = subset(EMMunpprint,  Temperature == '47.1°C' & Medication == 'Oxytocin' & Emo=='negative'),
          aes( y = c(infomax,infomax), x=c(1.765,2.235)),color="#999999")+
  geom_text(data = subset(EMMunpprint,  Temperature == '47.1°C' & Medication == 'Oxytocin' & Emo=='negative'),
          aes( y = infomax-8, x=2, label='*'), size = 8,color="#999999")+
  guides(fill=FALSE)+
  facet_grid( ~ Temperature)+   #Split-Grid by Temperature
  #Adds Means and Error-Bars for each Condition
  geom_point(data=EMMunpprint,    aes(x = Medication, y = lsmean, group=Emo, color=Emo),
position=position_dodge(width=0.7),  size = 3)+
  geom_errorbar(data=EMMunpprint, aes(x = Medication, y = lsmean, group=Emo, color=Emo, ymax = upper.CL, ymin =
lower.CL),position=position_dodge(width=0.7),width=0,  size = 1.5)+
  scale_colour_manual("Emotion:",values = c("#0072B2", "#666666", "#CC79A7"),labels= c("negative", "neutral",
"positive"))+   #Change Legend Nameing and Position
  scale_y_continuous("Heat unpleasantness rating")+ #Change axis legend
  theme(legend.position='bottom',legend.direction
="horizontal",legend.text=element_text(size=12),legend.title=element_text(size=14,face="bold"),legend.key=element_blank
())+
  ggtitle("b)")+theme(plot.title = element_text(hjust = 0))+
  theme(aspect.ratio = 1/sqrt(2))+ #fix plot size
  theme(axis.text=element_text(size=12), #fix axis font size
       axis.title=element_text(size=14,face="bold"))+ #fix axis title font size
  theme(strip.text.x = element_text(size = 12,face="bold"))+
  theme(axis.line = element_line(colour = "black"),
       panel.grid.major.y = element_line(colour = "#111111",size=0.2), #element_line(colour = "#555555"), #fix grid display
       panel.grid.minor.y = element_blank(), #fix grid display
       panel.grid.major.x = element_blank(),
       axis.title.x= element_blank(),
       panel.grid.minor = element_blank(),
       strip.background = element_rect(fill = 'transparent'),
       panel.background = element_blank())
unpplot
ggsave(
  "Figure3b.pdf",
  plot = unpplot,
  unit=c("mm"),
  width = gwidth,
  height = gheight,
  scale=3
)

#### ANALYSIS ZMVPA PERFORMANCE####
# # Check performance by OVERALL CORRELATION:
```

```
cor(as.double(Oxy2$Temperature),Oxy2$MVPA,use="na.or.complete") # Correlations for all conditions
CIr(cor(as.double(Oxy2$Temperature),Oxy2$MVPA,use="na.or.complete"), n = length(Oxy2$MVPA), level = .95)
plot(as.double(Oxy2$Temperature),Oxy2$MVPA)

cor(as.double(Oxy2$Intensity),Oxy2$MVPA,use="na.or.complete") # Correlations for all conditions
CIr(cor(as.double(Oxy2$Intensity),Oxy2$MVPA,use="na.or.complete"), n = length(Oxy2$MVPA), level = .95)
plot(Oxy2$Intensity,Oxy2$MVPA)

cor(as.double(Oxy2$Unpleasantness),Oxy2$MVPA,use="na.or.complete") # Correlations for all conditions
CIr(cor(as.double(Oxy2$Unpleasantness),Oxy2$MVPA,use="na.or.complete"), n = length(Oxy2$MVPA), level = .95)
plot(Oxy2$Unpleasantness,Oxy2$MVPA)

cor(as.double(Oxy2$Intensity),Oxy2$MVPA,use="na.or.complete") # Correlations for all conditions
CIr(cor(as.double(Oxy2$Intensity),Oxy2$MVPA,use="na.or.complete"), n = length(Oxy2$MVPA), level = .95)

# Check performance by DICHOTOMIZATION:
# Check Accuracy (TruePos+TrueNeg/Total)
Oxy2$bySubjmedianMVPA<-ave(Oxy2$MVPA,Oxy2$Proband,FUN=median,na.rm=TRUE) # Calculate median NPS value for each participant
Oxy2$TempClassfromMVPA<-(Oxy2$MVPA-Oxy2$bySubjmedianMVPA)>0     # Dichotomize: Values >0 represent pain, values =<0 Nopain
Oxy2$CorrClassbyMVPA<-(Oxy2$TempClassfromMVPA) == (Oxy2$Temperature=="47.1°C") # Which NPS value >0 match the high-temperature condition
percentCorrectMVPA=sum(Oxy2$CorrClassbyMVPA[Oxy2$Temperature!="35.0°C"],na.rm=TRUE)/length(Oxy2$CorrClassbyMVPA[Oxy2$Temperature!="35.0°C"]) # % Correct for 44.7 and 47.1°C
percentCorrectMVPA #Accuracy
# Check Precision
Oxy2$CorrPosMVPA<-(Oxy2$TempClassfromMVPA==1 & Oxy2$Temperature=="47.1°C")
Oxy2$FalsePosMVPA<-(Oxy2$TempClassfromMVPA==1 & Oxy2$Temperature!="47.1°C")
precisionMVPA=sum(Oxy2$CorrPosMVPA,na.rm=TRUE)/(sum(Oxy2$CorrPosMVPA,na.rm=TRUE)+sum(Oxy2$FalsePosMVPA,na.rm=TRUE)) # % Correct for 44.7 and 47.1°C
precisionMVPA
# Check Accuracy Intensity (TruePos+TrueNeg/Total)
Oxy2$bySubjmedianIntensity<-ave(Oxy2$Intensity,Oxy2$Proband,FUN=median,na.rm=TRUE) # Calculate median NPS value for each participant
Oxy2$TempClassfromIntensity<-(Oxy2$Intensity-Oxy2$bySubjmedianIntensity)>0     # Dichotomize: Values >0 represent pain, values =<0 Nopain
Oxy2$CorrClassbyIntensity<-(Oxy2$TempClassfromIntensity) == (Oxy2$Temperature=="47.1°C") # Which NPS value >0 match the high-temperature condition
percentCorrectIntensity=sum(Oxy2$CorrClassbyIntensity[Oxy2$Temperature!="35.0°C"],na.rm=TRUE)/length(Oxy2$CorrClassbyIntensity[Oxy2$Temperature!="35.0°C"]) # % Correct for 44.7 and 47.1°C
percentCorrectIntensity
# Check Precision Intensity
Oxy2$CorrPosInt<-(Oxy2$TempClassfromIntensity==1 & Oxy2$Temperature=="47.1°C")
Oxy2$FalsePosInt<-(Oxy2$TempClassfromIntensity==1 & Oxy2$Temperature!="47.1°C")
```

```
precisionInt=sum(Oxy2$CorrPosInt,na.rm=TRUE)/(sum(Oxy2$CorrPosInt,na.rm=TRUE)+sum(Oxy2$FalsePosInt,na.rm=T
RUE)) # % Correct for 44.7 and 47.1°C
precisionInt
# Check Accuracy Unpleasantness (TruePos+TrueNeg/Total)
Oxy2$bySubjmedianUnpleasantness<-ave(Oxy2$Unpleasantness,Oxy2$Proband,FUN=median,na.rm=TRUE) # Calculate
median NPS value for each participant
Oxy2$TempClassfromUnpleasantness<-(Oxy2$Unpleasantness-Oxy2$bySubjmedianUnpleasantness)>0     # Dichotomize:
Values >0 represent pain, values =<0 Nopain
Oxy2$CorrClassbyUnpleasantness<-(Oxy2$TempClassfromUnpleasantness) == (Oxy2$Temperature=="47.1°C") # Which
NPS value >0 match the high-temperature condition
percentCorrectUnpleasantness=sum(Oxy2$CorrClassbyUnpleasantness[Oxy2$Temperature!="35.0°C"],na.rm=TRUE)/leng
th(Oxy2$CorrClassbyUnpleasantness[Oxy2$Temperature!="35.0°C"]) # % Correct for 44.7 and 47.1°C
percentCorrectUnpleasantness
# Check Precision Unpleasantness
Oxy2$CorrPosUnp<-(Oxy2$TempClassfromUnpleasantness==1 & Oxy2$Temperature=="47.1°C")
Oxy2$FalsePosUnp<-(Oxy2$TempClassfromUnpleasantness==1 & Oxy2$Temperature!="47.1°C")
precisionUnp=sum(Oxy2$CorrPosUnp,na.rm=TRUE)/(sum(Oxy2$CorrPosUnp,na.rm=TRUE)+sum(Oxy2$FalsePosUnp,na
.rm=TRUE)) # % Correct for 44.7 and 47.1°C
precisionUnp

#### ANALYSIS MVPA MAIN ####
#  MVPA (Standardized)
convergenceScaling=1 # Scaling factor necessary for some variables to achieve convergence in gradient descent
# Calculate Model
MM_MVPA_full<-lmer(ZMVPA*convergenceScaling~(Medication+Temperature+Emo)^3+
                  (1+(Medication+Temperature+Emo)^2|Proband),
                data=Oxywo35woScr,REML=TRUE, control=lmerControl(optCtrl=list(maxfun=180000)),na.action =na.omit)
# Generates Summary Output for Model
summary(MM_MVPA_full)
# Generates ANOVA Statistiv via lmerTest
anova(MM_MVPA_full,ddf = "Kenward-Roger")

# Estimate Marginal Means using lsmeans by Russell V. Lenth The University of Iowa October 9, 2015
gridMVPA=ref.grid(MM_MVPA_full) # Create the reference grid for all comparisons
#Estimate Marginal Means for TEMPERATURE
EMMMVPATemp<- lsmeans(gridMVPA,~Temperature)
CMVPA.Temp<-pairs(EMMMVPATemp) #Test pairwise differences
CMVPA.Temp.Results<-show(CMVPA.Temp)
#Standardized Effect
CMVPA.Temp.ZResults<-CMVPA.Temp.Results
CMVPA.Temp.ZResults$estimate<-CMVPA.Temp.ZResults$estimate/convergenceScaling # Re-Scale contrast estimates to
SD
CMVPA.Temp.ZResults$SE<-CMVPA.Temp.ZResults$SE/convergenceScaling
CMVPA.Temp.ZResults$CIlo<-CMVPA.Temp.ZResults$estimate-(CMVPA.Temp.ZResults$SE*qt(.975,
df=CMVPA.Temp.ZResults$df))
```

CMVPA.Temp.ZResults$CIhi<-CMVPA.Temp.ZResults$estimate+(CMVPA.Temp.ZResults$SE*qt(.975, df=CMVPA.Temp.ZResults$df))
CMVPA.Temp.ZResults
#Non-Standardized Effect
CMVPA.Temp.Results$estimate<-
CMVPA.Temp.Results$estimate*sd(Oxywo35woScr$MVPA,na.rm=TRUE)/convergenceScaling # Re-Scale contrast estimates and CI's from z-Scores to raw Scaling
CMVPA.Temp.Results$SE<-CMVPA.Temp.Results$SE*sd(Oxywo35woScr$MVPA,na.rm=TRUE)/convergenceScaling
CMVPA.Temp.Results$CIlo<-CMVPA.Temp.Results$estimate-(CMVPA.Temp.Results$SE*qt(.975, df=CMVPA.Temp.Results$df))
CMVPA.Temp.Results$CIhi<-CMVPA.Temp.Results$estimate+(CMVPA.Temp.Results$SE*qt(.975, df=CMVPA.Temp.Results$df))
CMVPA.Temp.Results
#Estimate Marginal Means for EMOTION
EMMMVPAEmo<- lsmeans(gridMVPA,~Emo)
CMVPA.Emo<-pairs(EMMMVPAEmo) #Test pairwise differences.
CMVPA.Emo.Results<-show(CMVPA.Emo)
#Standardized Effect
CMVPA.Emo.ZResults<-CMVPA.Emo.Results
CMVPA.Emo.ZResults$estimate<-CMVPA.Emo.ZResults$estimate/convergenceScaling # Re-Scale contrast estimates to SD
CMVPA.Emo.ZResults$SE<-CMVPA.Emo.ZResults$SE/convergenceScaling
CMVPA.Emo.ZResults$CIlo<-CMVPA.Emo.ZResults$estimate-(CMVPA.Emo.ZResults$SE*qt(.975, df=CMVPA.Emo.ZResults$df))
CMVPA.Emo.ZResults$CIhi<-CMVPA.Emo.ZResults$estimate+(CMVPA.Emo.ZResults$SE*qt(.975, df=CMVPA.Emo.ZResults$df))
CMVPA.Emo.ZResults
#Non-Standardized Effect
CMVPA.Emo.Results$estimate<-
CMVPA.Emo.Results$estimate*sd(Oxywo35woScr$MVPA,na.rm=TRUE)/convergenceScaling # Re-Scale contrast estimates and CI's from z-Scores to raw Scaling
CMVPA.Emo.Results$SE<-CMVPA.Emo.Results$SE*sd(Oxywo35woScr$MVPA,na.rm=TRUE)/convergenceScaling
CMVPA.Emo.Results$CIlo<-CMVPA.Emo.Results$estimate-(CMVPA.Emo.Results$SE*qt(.975, df=CMVPA.Emo.Results$df))
CMVPA.Emo.Results$CIhi<-CMVPA.Emo.Results$estimate+(CMVPA.Emo.Results$SE*qt(.975, df=CMVPA.Emo.Results$df))
CMVPA.Emo.Results
#Estimate Marginal Means for MEDICATION:EMOTION
EMMMVPAMedEmo<- lsmeans(gridMVPA,~Medication:Emo)
CMVPA.MedEmo<-pairs(EMMMVPAMedEmo)
CMVPA.MedEmo.Results<-show(CMVPA.MedEmo)
#Standardized Effect
CMVPA.MedEmo.ZResults<-CMVPA.MedEmo.Results
CMVPA.MedEmo.ZResults$estimate<-CMVPA.MedEmo.ZResults$estimate/convergenceScaling # Re-Scale contrast estimates to SD
CMVPA.MedEmo.ZResults$SE<-CMVPA.MedEmo.ZResults$SE/convergenceScaling

```
CMVPA.MedEmo.ZResults$CIlo<-CMVPA.MedEmo.ZResults$estimate-(CMVPA.MedEmo.ZResults$SE*qt(.975,
df=CMVPA.MedEmo.ZResults$df))
CMVPA.MedEmo.ZResults$CIhi<-CMVPA.MedEmo.ZResults$estimate+(CMVPA.MedEmo.ZResults$SE*qt(.975,
df=CMVPA.MedEmo.ZResults$df))
CMVPA.MedEmo.ZResults
#Non-Standardized Effect
CMVPA.MedEmo.Results$estimate<-
CMVPA.MedEmo.Results$estimate*sd(Oxywo35woScr$MVPA,na.rm=TRUE)/convergenceScaling # Re-Scale contrast
estimates and CI's from z-Scores to raw Scaling
CMVPA.MedEmo.Results$SE<-
CMVPA.MedEmo.Results$SE*sd(Oxywo35woScr$MVPA,na.rm=TRUE)/convergenceScaling
CMVPA.MedEmo.Results$CIlo<-CMVPA.MedEmo.Results$estimate-(CMVPA.MedEmo.Results$SE*qt(.975,
df=CMVPA.MedEmo.Results$df))
CMVPA.MedEmo.Results$CIhi<-CMVPA.MedEmo.Results$estimate+(CMVPA.MedEmo.Results$SE*qt(.975,
df=CMVPA.MedEmo.Results$df))
CMVPA.MedEmo.Results
#Estimate Marginal Means for MEDICATION:TEMPERATURE
EMMMVPA<- lsmeans(gridMVPA,~Medication:Temperature:Emo)
CMVPA.3way<-pairs(EMMMVPA)
CMVPA.3way.results<-show(CMVPA.3way)
CMVPA.3way.Zresults<-CMVPA.3way.results
#Standardized Effect
CMVPA.3way.Zresults$estimate<-CMVPA.3way.Zresults$estimate/convergenceScaling
CMVPA.3way.Zresults$SE<-CMVPA.3way.Zresults$SE/convergenceScaling
CMVPA.3way.Zresults$CIlo<-CMVPA.3way.Zresults$estimate-(CMVPA.3way.Zresults$SE*qt(.975,
df=CMVPA.3way.Zresults$df))
CMVPA.3way.Zresults$CIhi<-CMVPA.3way.Zresults$estimate+(CMVPA.3way.Zresults$SE*qt(.975,
df=CMVPA.3way.Zresults$df))
CMVPA.3way.Zresults
#Unstandardized Effect
CMVPA.3way.results$estimate<-
CMVPA.3way.results$estimate*sd(Oxywo35woScr$MVPA,na.rm=TRUE)/convergenceScaling
CMVPA.3way.results$SE<-CMVPA.3way.results$SE*sd(Oxywo35woScr$MVPA,na.rm=TRUE)/convergenceScaling
CMVPA.3way.results$CIlo<-CMVPA.3way.results$estimate-(CMVPA.3way.results$SE*qt(.975,
df=CMVPA.3way.results$df))
CMVPA.3way.results$CIhi<-CMVPA.3way.results$estimate+(CMVPA.3way.results$SE*qt(.975,
df=CMVPA.3way.results$df))
CMVPA.3way.results
# Estimate Marginal Means for Plot
EMMMVPAprint<-show(EMMMVPA)
# Backtransform standardized estimates to actual ratings
EMMMVPAprint$lsmean<-
EMMMVPAprint$lsmean*sd(Oxywo35woScr$MVPA,na.rm=TRUE)/convergenceScaling+mean(Oxywo35woScr$MVPA,
na.rm=TRUE) #Transform EMM back to raw scale (inverse z-Transform)
EMMMVPAprint$SE<-EMMMVPAprint$SE*sd(Oxywo35woScr$MVPA,na.rm=TRUE)/convergenceScaling
```

```
EMMMVPAprint$lower.CL<-
EMMMVPAprint$lower.CL*sd(Oxywo35woScr$MVPA,na.rm=TRUE)/convergenceScaling+mean(Oxywo35woScr$MVP
A,na.rm=TRUE)
EMMMVPAprint$upper.CL<-
EMMMVPAprint$upper.CL*sd(Oxywo35woScr$MVPA,na.rm=TRUE)/convergenceScaling+mean(Oxywo35woScr$MVP
A,na.rm=TRUE)

#Plot MVPA
MVPAplot=ggplot() +
  geom_jitter(data=Oxywo35woScr,     aes(x = Medication, y = MVPA, group=Emo, color=Emo,fill = Emo),
position=position_jitterdodge(jitter.width = 0.2,dodge.width=0.7),alpha=0.2) +   #Single-Value Dots
  guides(fill=FALSE)+
  facet_grid( ~ Temperature)+   #Split-Grid by Temperature
  #Adds Means and Error-Bars for each Condition
  geom_point(data=EMMMVPAprint,    aes(x = Medication, y = lsmean, group=Emo, color=Emo),
position=position_dodge(width=0.7),  size = 3)+
  geom_errorbar(data=EMMMVPAprint, aes(x = Medication, y = lsmean, group=Emo, color=Emo, ymax = upper.CL, ymin
= lower.CL),position=position_dodge(width=0.7),width=0,  size = 1.5)+
  scale_colour_manual("Emotion:",values = c("#0072B2", "#666666", "#CC79A7"),labels= c("negative", "neutral",
"positive"))+   #Change Legend Nameing and Position
  scale_y_continuous("MHT")+ #Change axis legend
  theme(legend.position='bottom',legend.direction
="horizontal",legend.text=element_text(size=12),legend.title=element_text(size=14,face="bold"),legend.key=element_blank
())+
  ggtitle("c)")+theme(plot.title = element_text(hjust = 0))+
  theme(aspect.ratio = 1/sqrt(2))+ #fix plot size
  theme(axis.text=element_text(size=12), #fix axis font size
        axis.title=element_text(size=14,face="bold"))+ #fix axis title font size
  theme(strip.text.x = element_text(size = 12,face="bold"))+
  theme(panel.grid.major.y = element_line(colour = "#111111",size=0.2), #fix grid display
        panel.grid.minor.y = element_blank(), #fix grid display
        panel.grid.major.x = element_blank(),
        axis.title.x= element_blank(),
        panel.grid.minor = element_blank(),
        strip.background = element_rect(fill = 'transparent'),
        panel.background = element_blank(), axis.line = element_line(colour = "black"))
MVPAplot
ggsave(
  "Figure3c.pdf",
  plot = MVPAplot,
  unit=c("mm"),
  width = gwidth,
  height = gheight,
  scale=3
)
```

```
#### Analysis of SCRAMBLED CONTROL
#### SELECT DATA FOR SCRAMBLED CONTROL (Scrambled excluding 35°C)
Oxywo35<-subset(Oxy2, Temperature != "35.0°C") #
Oxywo35$Temperature<-factor(Oxywo35$Temperature)
Oxywo35$Scr<-factor(as.integer(Oxywo35$Emo=='scrambled'))
Oxywo35$ZIntensity=as.numeric(scale(Oxywo35$Intensity, center = TRUE, scale = TRUE))
Oxywo35$ZUnpleasantness=as.numeric(scale(Oxywo35$Unpleasantness, center = TRUE, scale = TRUE))
Oxywo35$ZMVPA=as.numeric(scale(Oxywo35$MVPA, center = TRUE, scale = TRUE))
#### ANALYSIS INTENSITY SCRAMBLED CONTROL ####
convergenceScaling=1 #Scale Values to Achieve Convergence in Mixed Model Estimation
MM_int_scr<-lmer(ZIntensity*convergenceScaling ~ (Medication+Temperature+Scr)^3
        +(1+(Medication+Temperature+Scr)^2|Proband),
        data=Oxywo35,REML=TRUE, control=lmerControl(optCtrl=list(maxfun=180000)),na.action =na.omit)
summary(MM_int_scr)
anova(MM_int_scr,ddf = "Kenward-Roger")

# Pairwise
gridIntScr=ref.grid(MM_int_scr) # Create the reference grid for comparisons
# for Intensity Scr ... see: "Using lsmeans" by Russell V. Lenth The University of Iowa October 9, 2015
EMMintScrScr<- lsmeans(gridIntScr,~Scr)
CInt.Scr<-pairs(EMMintScrScr)
CInt.Scr.Results<-show(CInt.Scr)
CInt.Scr.Results$estimate<-CInt.Scr.Results$estimate*sd(Oxywo35$Intensity,na.rm=TRUE)/convergenceScaling
CInt.Scr.Results$SE<-CInt.Scr.Results$SE*sd(Oxywo35$Intensity,na.rm=TRUE)/convergenceScaling
CInt.Scr.Results$CIlo<-CInt.Scr.Results$estimate-(CInt.Scr.Results$SE*qt(.975, df=CInt.Scr.Results$df))
CInt.Scr.Results$CIhi<-CInt.Scr.Results$estimate+(CInt.Scr.Results$SE*qt(.975, df=CInt.Scr.Results$df))
CInt.Scr.Results
#Plot ANALYSIS INTENSITY SCRAMBLED CONTROL
#Estimate Marginal Means for Plot
EMMintScr<- lsmeans(gridIntScr,~Medication:Temperature:Scr)
EMMintprintScr<-show(EMMintScr)
EMMintprintScr$lsmean<-
EMMintprintScr$lsmean*sd(Oxywo35$Intensity,na.rm=TRUE)/convergenceScaling+mean(Oxywo35$Intensity,na.rm=TR
UE) #Transform EMM back to raw scale (inverse z-Transform)
EMMintprintScr$SE<-EMMintprintScr$SE*sd(Oxywo35$Intensity,na.rm=TRUE)/convergenceScaling
EMMintprintScr$lower.CL<-
EMMintprintScr$lower.CL*sd(Oxywo35$Intensity,na.rm=TRUE)/convergenceScaling+mean(Oxywo35$Intensity,na.rm=T
RUE)
EMMintprintScr$upper.CL<-
EMMintprintScr$upper.CL*sd(Oxywo35$Intensity,na.rm=TRUE)/convergenceScaling+mean(Oxywo35$Intensity,na.rm=T
RUE)

intScrplot=ggplot() +
  geom_jitter(data=Oxywo35,     aes(x = Medication, y = Intensity, group=Scr, color=Scr,fill = Emo),
position=position_jitterdodge(jitter.width = 0.2,dodge.width=0.7),alpha=0.2) +   #Single-Value Dots
```

```
  guides(fill=FALSE)+
  facet_grid( ~ Temperature)+   #Split-Grid by Temperature
  # Show interaction±SE
  #Adds Means and Error-Bars for each Condition
  geom_point(data=EMMintprintScr,    aes(x = Medication, y = lsmean, group=Scr, color=Scr),
position=position_dodge(width=0.7),  size = 3)+
  geom_errorbar(data=EMMintprintScr, aes(x = Medication, y = lsmean, group=Scr, color=Scr, ymax = upper.CL, ymin =
lower.CL),position=position_dodge(width=0.7),width=0,  size = 1.5)+
  scale_colour_manual("Scrambled:",values = c("#0072B2", "#666666"),labels= c("Non-Scrambled", "Scrambled"))+
#Change Legend Nameing and Position
  scale_y_continuous("Heat intensity rating")+ #Change axis legend
  theme(legend.position='bottom',legend.direction
="horizontal",legend.text=element_text(size=12),legend.title=element_text(size=14,face="bold"),legend.key=element_blank
())+
  ggtitle("a)")+theme(plot.title = element_text(hjust = 0))+
  #theme(aspect.ratio = 1/sqrt(2))+ #fix plot size
  theme(axis.text=element_text(size=12), #fix axis font size
        axis.title=element_text(size=14,face="bold"))+ #fix axis title font size
  theme(strip.text.x = element_text(size = 12,face="bold"))+
  theme(panel.grid.major.y = element_line(colour = "#111111",size=0.2),
        panel.grid.minor.y = element_blank(), #fix grid display
        panel.grid.major.x = element_blank(),
        axis.title.x= element_blank(),
        panel.grid.minor = element_blank(),
        strip.background = element_rect(fill = 'transparent'),
        panel.background = element_blank(), axis.line = element_line(colour = "black"))
intScrplot
ggsave(
  "Figure4a.pdf",
  plot = intScrplot,
  unit=c("mm"),
  width = gwidth,
  height = gheight,
  scale=3
)

#### ANALYSIS UNPLEASANTNESS SCRAMBLED CONTROL####
convergenceScaling=1 #Scale Values to Achieve Convergence in Mixed Model Estimation
MM_unp_scr<-lmer(ZUnpleasantness*convergenceScaling ~ (Medication+Temperature+Scr)^3
              +(1+(Medication+Temperature+Scr)^2|Proband),
              data=Oxywo35,REML=TRUE, control=lmerControl(optCtrl=list(maxfun=180000)),na.action =na.omit)
summary(MM_unp_scr)
anova(MM_unp_scr,ddf = "Kenward-Roger") #

gridUnpScr=ref.grid(MM_unp_scr) # Create the reference grid for comparisons
EMMunpScrScr<- lsmeans(gridUnpScr,~Scr)
```

```
CUnp.Scr<-pairs(EMMunpScrScr)
CUnp.Scr.Results<-show(CUnp.Scr)
CUnp.Scr.ZResults<-CUnp.Scr.Results
# Standardized
CUnp.Scr.ZResults$estimate<-CUnp.Scr.ZResults$estimate/convergenceScaling
CUnp.Scr.ZResults$SE<-CUnp.Scr.ZResults$SE/convergenceScaling
CUnp.Scr.ZResults$CIlo<-CUnp.Scr.ZResults$estimate-(CUnp.Scr.ZResults$SE*qt(.975, df=CUnp.Scr.ZResults$df))
CUnp.Scr.ZResults$CIhi<-CUnp.Scr.ZResults$estimate+(CUnp.Scr.ZResults$SE*qt(.975, df=CUnp.Scr.ZResults$df))
CUnp.Scr.ZResults
# Unstandardized
CUnp.Scr.Results$estimate<-CUnp.Scr.Results$estimate*sd(Oxywo35$Unpleasantness,na.rm=TRUE)/convergenceScaling
CUnp.Scr.Results$SE<-CUnp.Scr.Results$SE*sd(Oxywo35$Unpleasantness,na.rm=TRUE)/convergenceScaling
CUnp.Scr.Results$CIlo<-CUnp.Scr.Results$estimate-(CUnp.Scr.Results$SE*qt(.975, df=CUnp.Scr.Results$df))
CUnp.Scr.Results$CIhi<-CUnp.Scr.Results$estimate+(CUnp.Scr.Results$SE*qt(.975, df=CUnp.Scr.Results$df))
CUnp.Scr.Results
#No effects of scrambled and oxytocin >> no plot

#### ANALYSIS MVPA SCRAMBLED CONTROL w Graph ####
convergenceScaling=100 #Scale Values to Achieve Convergence in Mixed Model Estimation
MM_MVPA_scr<-lmer(ZMVPA*convergenceScaling ~ (Medication+Temperature+Scr)^3
        +(1+(Medication+Temperature+Scr)^2|Proband),
        data=Oxywo35,REML=TRUE, control=lmerControl(optCtrl=list(maxfun=180000)),na.action =na.omit)
summary(MM_MVPA_scr)
anova(MM_MVPA_scr,ddf = "Kenward-Roger") #
gridMVPAscr=ref.grid(MM_MVPA_scr) # Create the reference grid for comparisons

# Pairwise for MVPA Scr ... see: "Using lsmeans" by Russell V. Lenth The University of Iowa October 9, 2015
EMMMVPAscrscr<- lsmeans(gridMVPAscr,~Scr)
CMVPA.Scr<-pairs(EMMMVPAscrscr)
CMVPA.Scr.Results<-show(CMVPA.Scr)
CMVPA.Scr.ZResults<-CMVPA.Scr.Results
# Standardized
CMVPA.Scr.ZResults$estimate<-CMVPA.Scr.ZResults$estimate/convergenceScaling
CMVPA.Scr.ZResults$SE<-CMVPA.Scr.ZResults$SE/convergenceScaling
CMVPA.Scr.ZResults$CIlo<-CMVPA.Scr.ZResults$estimate-(CMVPA.Scr.ZResults$SE*qt(.975,
df=CMVPA.Scr.ZResults$df))
CMVPA.Scr.ZResults$CIhi<-CMVPA.Scr.ZResults$estimate+(CMVPA.Scr.ZResults$SE*qt(.975,
df=CMVPA.Scr.ZResults$df))
CMVPA.Scr.ZResults
# Unstandardized
CMVPA.Scr.Results$estimate<-CMVPA.Scr.Results$estimate*sd(Oxywo35$MVPA,na.rm=TRUE)/convergenceScaling
CMVPA.Scr.Results$SE<-CMVPA.Scr.Results$SE*sd(Oxywo35$MVPA,na.rm=TRUE)/convergenceScaling
CMVPA.Scr.Results$CIlo<-CMVPA.Scr.Results$estimate-(CMVPA.Scr.Results$SE*qt(.975, df=CMVPA.Scr.Results$df))
CMVPA.Scr.Results$CIhi<-CMVPA.Scr.Results$estimate+(CMVPA.Scr.Results$SE*qt(.975,
df=CMVPA.Scr.Results$df))
CMVPA.Scr.Results
```

```
# Compare Scr Effect vs Emo Effect
CMVPA.Scr.Results$estimate/CMVPA.Emo.Results$estimate[1]

#Plot MVPA
#Estimate Marginal Means for Plot
EMMMVPAscr<- lsmeans(gridMVPAscr,~Medication:Temperature:Scr)
EMMMVPAscrprint<-show(EMMMVPAscr)
EMMMVPAscrprint$lsmean<-
EMMMVPAscrprint$lsmean*sd(Oxywo35$MVPA,na.rm=TRUE)/convergenceScaling+mean(Oxywo35$MVPA,na.rm=TR
UE) #Transform EMM back to raw scale (inverse z-Transform)
EMMMVPAscrprint$SE<-EMMMVPAscrprint$SE*sd(Oxywo35$MVPA,na.rm=TRUE)/convergenceScaling
EMMMVPAscrprint$lower.CL<-
EMMMVPAscrprint$lower.CL*sd(Oxywo35$MVPA,na.rm=TRUE)/convergenceScaling+mean(Oxywo35$MVPA,na.rm=
TRUE)
EMMMVPAscrprint$upper.CL<-
EMMMVPAscrprint$upper.CL*sd(Oxywo35$MVPA,na.rm=TRUE)/convergenceScaling+mean(Oxywo35$MVPA,na.rm=
TRUE)
MVPAplotscr=ggplot() +
  geom_jitter(data=Oxywo35,       aes(x = Medication, y = MVPA, group=Scr, color=Scr, ymax = max(MVPA),fill = Emo),
position=position_jitterdodge(jitter.width = 0.2,dodge.width=0.7),alpha=0.2) +   #Single-Value Dots
  guides(fill=FALSE)+
  facet_grid( ~ Temperature)+   #Split-Grid by Temperature
  # Show interaction±SE
  #Adds Means and Error-Bars for each Condition
  geom_point(data=EMMMVPAscrprint,    aes(x = Medication, y = lsmean, group=Scr, color=Scr, ymax = max(lsmean)),
position=position_dodge(width=0.7),  size = 3)+
  geom_errorbar(data=EMMMVPAscrprint, aes(x = Medication, y = lsmean, group=Scr, color=Scr, ymax = upper.CL, ymin
= lower.CL),position=position_dodge(width=0.7),width=0,  size = 1.5)+
  scale_colour_manual("Scrambled:",values = c("#0072B2", "#666666"),labels= c("Non-Scrambled", "Scrambled"))+
#Change Legend Nameing and Position
  scale_y_continuous("MHT")+ #Change axis legend
  labs(title = "b")+theme(plot.title = element_text(hjust = 0))+
  theme(legend.position='bottom',legend.direction
="horizontal",legend.text=element_text(size=12),legend.title=element_text(size=14,face="bold"),legend.key=element_blank
())+
  theme(aspect.ratio = 1/sqrt(2))+ #fix plot size
  theme(axis.text=element_text(size=12), #fix axis font size
       axis.title=element_text(size=14,face="bold"))+ #fix axis title font size
  theme(strip.text.x = element_text(size = 12,face="bold"))+
  theme(panel.grid.major.y = element_line(colour = "#111111",size=0.2), #fix grid display
       panel.grid.minor.y = element_blank(), #fix grid display
       panel.grid.major.x = element_blank(),
       axis.title.x= element_blank(),
       panel.grid.minor = element_blank(),
       strip.background = element_rect(fill = 'transparent'),
       panel.background = element_blank(), axis.line = element_line(colour = "black"))
```

```
MVPAplotscr
ggsave(
  "Figure4b.pdf",
  plot = MVPAplotscr,
  unit=c("mm"),
  width = gwidth,
  height = gheight,
  scale=3
)

#### BASELINE TEMPERATURE CONTROL####
#### SELECT DATA FOR BASELINE TEMPERATURE CONTROL (35°C, WO Scrambled) ####
Oxy35only<-subset(Oxy2, Temperature == "35.0°C" & Emo != 'scrambled') #
Oxy35only$Emo<-factor(Oxy35only$Emo) #Reassign factors to remove factor levels
Oxy35only$Scr<-factor(as.integer(Oxy35only$Emo=='scrambled'))
Oxy35only$Temperature<-factor(Oxy35only$Temperature) #Reassign factors to remove factor levels
Oxy35only$ZIntensity=as.numeric(scale(Oxy35only$Intensity, center = TRUE, scale = TRUE))
Oxy35only$ZUnpleasantness=as.numeric(scale(Oxy35only$Unpleasantness, center = TRUE, scale = TRUE))
Oxy35only$ZMVPA=as.numeric(scale(Oxy35only$MVPA, center = TRUE, scale = TRUE))

#### ANALYSIS INTENSITY 35°C CONTROL ####
sum(Oxy35only$Intensity<5)/length(Oxy35only$Intensity)
hist(Oxy35only$Intensity)
# Results near 0, using pairwise non-parametric statistics to explore
Test35=Oxy35only$Medication:Oxy35only$Emo
wilcox.test(Oxy35only$Intensity[Test35=="Placebo:negative"], Oxy35only$Intensity[Test35=="Placebo:neutral"],
paired=TRUE)
wilcox.test(Oxy35only$Intensity[Test35=="Placebo:negative"], Oxy35only$Intensity[Test35=="Placebo:positive"],
paired=TRUE)
wilcox.test(Oxy35only$Intensity[Test35=="Placebo:neutral"], Oxy35only$Intensity[Test35=="Placebo:positive"],
paired=TRUE)
wilcox.test(Oxy35only$Intensity[Test35=="Oxytocin:negative"], Oxy35only$Intensity[Test35=="Oxytocin:neutral"],
paired=TRUE)
wilcox.test(Oxy35only$Intensity[Test35=="Oxytocin:negative"], Oxy35only$Intensity[Test35=="Oxytocin:positive"],
paired=TRUE)
wilcox.test(Oxy35only$Intensity[Test35=="Oxytocin:neutral"], Oxy35only$Intensity[Test35=="Oxytocin:positive"],
paired=TRUE)
wilcox.test(Oxy35only$Intensity[Test35=="Oxytocin:negative"], Oxy35only$Intensity[Test35=="Placebo:neutral"],
paired=TRUE)
wilcox.test(Oxy35only$Intensity[Test35=="Oxytocin:negative"], Oxy35only$Intensity[Test35=="Placebo:positive"],
paired=TRUE)
wilcox.test(Oxy35only$Intensity[Test35=="Oxytocin:neutral"], Oxy35only$Intensity[Test35=="Placebo:positive"],
paired=TRUE)
wilcox.test(Oxy35only$Intensity[Test35=="Oxytocin:negative"], Oxy35only$Intensity[Test35=="Placebo:negative"],
paired=TRUE)
```

```
wilcox.test(Oxy35only$Intensity[Test35=="Oxytocin:positive"], Oxy35only$Intensity[Test35=="Placebo:positive"],
paired=TRUE)
wilcox.test(Oxy35only$Intensity[Test35=="Oxytocin:neutral"], Oxy35only$Intensity[Test35=="Placebo:neutral"],
paired=TRUE)

#### ANALYSIS UNPLEASANTNESS 35°C CONTROL ####
sum(Oxy35only$Unpleasantness<5)/length(Oxy35only$Unpleasantness)
hist(Oxy35only$Unpleasantness)
# Results near 0, using pairwise non-parametric statistics to explore
Test35=Oxy35only$Medication:Oxy35only$Emo
wilcox.test(Oxy35only$Unpleasantness[Test35=="Placebo:negative"],
Oxy35only$Unpleasantness[Test35=="Placebo:neutral"], paired=TRUE)
wilcox.test(Oxy35only$Unpleasantness[Test35=="Placebo:negative"],
Oxy35only$Unpleasantness[Test35=="Placebo:positive"], paired=TRUE)
wilcox.test(Oxy35only$Unpleasantness[Test35=="Placebo:neutral"],
Oxy35only$Unpleasantness[Test35=="Placebo:positive"], paired=TRUE)
wilcox.test(Oxy35only$Unpleasantness[Test35=="Oxytocin:negative"],
Oxy35only$Unpleasantness[Test35=="Oxytocin:neutral"], paired=TRUE)
wilcox.test(Oxy35only$Unpleasantness[Test35=="Oxytocin:negative"],
Oxy35only$Unpleasantness[Test35=="Oxytocin:positive"], paired=TRUE)
wilcox.test(Oxy35only$Unpleasantness[Test35=="Oxytocin:neutral"],
Oxy35only$Unpleasantness[Test35=="Oxytocin:positive"], paired=TRUE)
wilcox.test(Oxy35only$Unpleasantness[Test35=="Oxytocin:negative"],
Oxy35only$Unpleasantness[Test35=="Placebo:neutral"], paired=TRUE)
wilcox.test(Oxy35only$Unpleasantness[Test35=="Oxytocin:negative"],
Oxy35only$Unpleasantness[Test35=="Placebo:positive"], paired=TRUE)
wilcox.test(Oxy35only$Unpleasantness[Test35=="Oxytocin:neutral"],
Oxy35only$Unpleasantness[Test35=="Placebo:positive"], paired=TRUE)
wilcox.test(Oxy35only$Unpleasantness[Test35=="Oxytocin:negative"],
Oxy35only$Unpleasantness[Test35=="Placebo:negative"], paired=TRUE)
wilcox.test(Oxy35only$Unpleasantness[Test35=="Oxytocin:positive"],
Oxy35only$Unpleasantness[Test35=="Placebo:positive"], paired=TRUE)
wilcox.test(Oxy35only$Unpleasantness[Test35=="Oxytocin:neutral"],
Oxy35only$Unpleasantness[Test35=="Placebo:neutral"], paired=TRUE)

#### ANALYSIS NPSzunClean 35°C CONTROL ####
convergenceScaling=1
# MVPA Analyze Temp 35 only
MM_MVPA_35<-lmer(ZMVPA*convergenceScaling~(Medication+Emo)^2+
            (1+(Medication+Emo)|Proband),
          data=Oxy35only,REML=TRUE, control=lmerControl(optCtrl=list(maxfun=180000)),na.action =na.omit)
#optimizer="Nelder_Mead",
summary(MM_MVPA_35)
anova(MM_MVPA_35,ddf = "Kenward-Roger")

gridMVPA35=ref.grid(MM_MVPA_35) # Create the reference grid for comparisons
```

```
EMMMVPAEmo<- lsmeans(gridMVPA35,~Emo)
CMVPA.35.Emo<-pairs(EMMMVPAEmo)
CMVPA.35.Emo.Results<-show(CMVPA.35.Emo)
CMVPA.35.Emo.ZResults<-CMVPA.35.Emo.Results
#Standardized
CMVPA.35.Emo.ZResults$estimate<-CMVPA.35.Emo.ZResults$estimate/convergenceScaling
CMVPA.35.Emo.ZResults$SE<-CMVPA.35.Emo.ZResults$SE/convergenceScaling
CMVPA.35.Emo.ZResults$CIlo<-CMVPA.35.Emo.ZResults$estimate-(CMVPA.35.Emo.ZResults$SE*qt(.975,
df=CMVPA.35.Emo.ZResults$df))
CMVPA.35.Emo.ZResults$CIhi<-CMVPA.35.Emo.ZResults$estimate+(CMVPA.35.Emo.ZResults$SE*qt(.975,
df=CMVPA.35.Emo.ZResults$df))
CMVPA.35.Emo.ZResults
#UnStandardized
CMVPA.35.Emo.Results$estimate<-
CMVPA.35.Emo.Results$estimate*sd(Oxywo35$MVPA,na.rm=TRUE)/convergenceScaling
CMVPA.35.Emo.Results$SE<-CMVPA.35.Emo.Results$SE*sd(Oxywo35$MVPA,na.rm=TRUE)/convergenceScaling
CMVPA.35.Emo.Results$CIlo<-CMVPA.35.Emo.Results$estimate-(CMVPA.35.Emo.Results$SE*qt(.975,
df=CMVPA.35.Emo.Results$df))
CMVPA.35.Emo.Results$CIhi<-CMVPA.35.Emo.Results$estimate+(CMVPA.35.Emo.Results$SE*qt(.975,
df=CMVPA.35.Emo.Results$df))
CMVPA.35.Emo.Results
#Estimate Marginal Means for Plot
EMMMVPA35<- lsmeans(gridMVPA35,~Medication:Emo)
EMMMVPA35print<-show(EMMMVPA35)
EMMMVPA35print$lsmean<-
EMMMVPA35print$lsmean*sd(Oxy35only$MVPA)/convergenceScaling+mean(Oxy35only$MVPA) #Transform EMM
back to raw scale (inverse z-Transform)
EMMMVPA35print$SE<-EMMMVPA35print$SE*sd(Oxy35only$MVPA,na.rm=TRUE)/convergenceScaling
EMMMVPA35print$lower.CL<-
EMMMVPA35print$lower.CL*sd(Oxy35only$MVPA,na.rm=TRUE)/convergenceScaling+mean(Oxy35only$MVPA,na.rm
=TRUE)
EMMMVPA35print$upper.CL<-
EMMMVPA35print$upper.CL*sd(Oxy35only$MVPA,na.rm=TRUE)/convergenceScaling+mean(Oxy35only$MVPA,na.rm
=TRUE)

#Plot MVPA baseline control
MVPAplot35=ggplot() +
  geom_jitter(data=Oxy35only,       aes(x = Medication, y = MVPA, group=Emo, color=Emo, ymax = max(MVPA),fill =
Emo), position=position_jitterdodge(jitter.width = 0.2,dodge.width=0.7),alpha=0.2) +   #Single-Value Dots
  guides(fill=FALSE)+
  facet_grid( ~ Temperature)+   #Split-Grid by Temperature
  # Show interaction±SE
  #Adds Means and Error-Bars for each Condition
  geom_point(data=EMMMVPA35print,    aes(x = Medication, y = lsmean, group=Emo, color=Emo, ymax = max(lsmean)),
position=position_dodge(width=0.7),  size = 3)+
```

```
  geom_errorbar(data=EMMMVPA35print, aes(x = Medication, y = lsmean, group=Emo, color=Emo, ymax = upper.CL,
ymin = lower.CL),position=position_dodge(width=0.7),width=0,  size = 1.5)+
  scale_colour_manual("Emotion:",values = c("#0072B2", "#666666","#CC79A7"),labels= c("negative", "neutral",
"positive"))+   #Change Legend Nameing and Position
  scale_y_continuous("MHT")+ #Change axis legend
  labs(title = "a")+theme(plot.title = element_text(hjust = 0))+
  theme(legend.position='bottom',legend.direction
="horizontal",legend.text=element_text(size=12),legend.title=element_text(size=14,face="bold"),legend.key=element_blank
())+
  theme(aspect.ratio = 1/sqrt(2))+ #fix plot size
  theme(axis.text=element_text(size=12), #fix axis font size
        axis.title=element_text(size=14,face="bold"))+ #fix axis title font size
  theme(strip.text.x = element_text(size = 12,face="bold"))+
  theme(panel.grid.major.y = element_line(colour = "#111111",size=0.2), #fix grid display
        panel.grid.minor.y = element_blank(), #fix grid display
        panel.grid.major.x = element_blank(),
        axis.title.x= element_blank(),
        panel.grid.minor = element_blank(),
        strip.background = element_rect(fill = 'transparent'),
        panel.background = element_blank(), axis.line = element_line(colour = "black"))
MVPAplot35
ggsave(
  "Figure5a.pdf",
  plot = MVPAplot35,
  unit=c("mm"),
  width = gwidth,
  height = gheight,
  scale=3
)

#### SELECT DATA FOR BASELINE TEMPERATURE CONTROL (35°C, w Scrambled) ####
Oxy35wScr<-subset(Oxy2, Temperature == "35.0°C") # & Emo != 'scrambled') #
Oxy35wScr$Emo<-factor(Oxy35wScr$Emo) #Reassign factors to remove factor levels
Oxy35wScr$Scr<-factor(as.integer(Oxy35wScr$Emo=='scrambled'))
Oxy35wScr$Temperature<-factor(Oxy35wScr$Temperature) #Reassign factors to remove factor levels
Oxy35wScr$ZIntensity=as.numeric(scale(Oxy35wScr$Intensity, center = TRUE, scale = TRUE))
Oxy35wScr$ZUnpleasantness=as.numeric(scale(Oxy35wScr$Unpleasantness, center = TRUE, scale = TRUE))
Oxy35wScr$ZMVPA=as.numeric(scale(Oxy35wScr$MVPA, center = TRUE, scale = TRUE))

#### ANALYSIS MVPA 35°C — SCRAMBLED ####
convergenceScaling=1
# MVPA Analyze Temp 35 only
MM_MVPA_35scr<-lmer(ZMVPA*convergenceScaling~(Medication+Scr)^2+
                 (1+(Medication+Scr)|Proband),
               data=Oxy35wScr,REML=TRUE, control=lmerControl(optCtrl=list(maxfun=180000)),na.action =na.omit)
#optimizer="Nelder_Mead",
```

```
summary(MM_MVPA_35scr)
anova(MM_MVPA_35scr,ddf = "Kenward-Roger")
gridMVPA35scr=ref.grid(MM_MVPA_35scr) # Create the reference grid for comparisons

#Pairwise
EMMMVPA.35.scrscr<- lsmeans(gridMVPA35scr,~Scr)
CMVPA.Scr35<-pairs(EMMMVPA.35.scrscr)
CMVPA.Scr35.Results<-show(CMVPA.Scr35)
CMVPA.Scr35.ZResults<-CMVPA.Scr35.Results
#Standardized
CMVPA.Scr35.ZResults$estimate<-CMVPA.Scr35.ZResults$estimate/convergenceScaling # Re-Scale contrast estimates and CI's from z-Scores to raw Scaling
CMVPA.Scr35.ZResults$SE<-CMVPA.Scr35.ZResults$SE/convergenceScaling
CMVPA.Scr35.ZResults$CIlo<-CMVPA.Scr35.ZResults$estimate-(CMVPA.Scr35.ZResults$SE*qt(.975, df=CMVPA.Scr35.ZResults$df))
CMVPA.Scr35.ZResults$CIhi<-CMVPA.Scr35.ZResults$estimate+(CMVPA.Scr35.ZResults$SE*qt(.975, df=CMVPA.Scr35.ZResults$df))
CMVPA.Scr35.ZResults
#Unstandardized
CMVPA.Scr35.Results$estimate<-
CMVPA.Scr35.Results$estimate*sd(Oxy35wScr$MVPA,na.rm=TRUE)/convergenceScaling # Re-Scale contrast estimates and CI's from z-Scores to raw Scaling
CMVPA.Scr35.Results$SE<-CMVPA.Scr35.Results$SE*sd(Oxy35wScr$MVPA,na.rm=TRUE)/convergenceScaling
CMVPA.Scr35.Results$CIlo<-CMVPA.Scr35.Results$estimate-(CMVPA.Scr35.Results$SE*qt(.975, df=CMVPA.Scr35.Results$df))
CMVPA.Scr35.Results$CIhi<-CMVPA.Scr35.Results$estimate+(CMVPA.Scr35.Results$SE*qt(.975, df=CMVPA.Scr35.Results$df))
CMVPA.Scr35.Results
#Estimate Marginal Means for Plot
EMMMVPA.35.scr<- lsmeans(gridMVPA35scr,~Medication:Scr)
EMMMVPA.35.scrprint<-show(EMMMVPA.35.scr)
EMMMVPA.35.scrprint$lsmean<-
EMMMVPA.35.scrprint$lsmean*sd(Oxy35wScr$MVPA,na.rm=TRUE)/convergenceScaling+mean(Oxy35wScr$MVPA,na.rm=TRUE) #Transform EMM back to raw scale (inverse z-Transform)
EMMMVPA.35.scrprint$SE<-EMMMVPA.35.scrprint$SE*sd(Oxy35wScr$MVPA,na.rm=TRUE)/convergenceScaling
EMMMVPA.35.scrprint$lower.CL<-
EMMMVPA.35.scrprint$lower.CL*sd(Oxy35wScr$MVPA,na.rm=TRUE)/convergenceScaling+mean(Oxy35wScr$MVPA, na.rm=TRUE)
EMMMVPA.35.scrprint$upper.CL<-
EMMMVPA.35.scrprint$upper.CL*sd(Oxy35wScr$MVPA,na.rm=TRUE)/convergenceScaling+mean(Oxy35wScr$MVPA, na.rm=TRUE)

MVPAplot35scr=ggplot() +
  geom_jitter(data=Oxy35wScr,      aes(x = Medication, y = MVPA, group=Scr, color=Scr, ymax = max(MVPA),fill = Scr), position=position_jitterdodge(jitter.width = 0.2,dodge.width=0.7),alpha=0.2) +   #Single-Value Dots
  guides(fill=FALSE)+
```

```
  facet_grid( ~ Temperature)+   #Split-Grid by Temperature
  # Show interaction±SE
  #Adds Means and Error-Bars for each Condition
  geom_point(data=EMMMVPA.35.scrprint,    aes(x = Medication, y = lsmean, group=Scr, color=Scr, ymax =
max(lsmean)),      position=position_dodge(width=0.7),  size = 3)+
  geom_errorbar(data=EMMMVPA.35.scrprint, aes(x = Medication, y = lsmean, group=Scr, color=Scr, ymax = upper.CL,
ymin = lower.CL),position=position_dodge(width=0.7),width=0,  size = 1.5)+
  scale_colour_manual("Scrambled:",values = c("#0072B2", "#666666"),labels= c("Non-Scrambled", "Scrambled"))+
#Change Legend Nameing and Position
  scale_y_continuous("MHT")+ #Change axis legend
  labs(title = "b")+theme(plot.title = element_text(hjust = 0))+
  theme(legend.position='bottom',legend.direction
="horizontal",legend.text=element_text(size=12),legend.title=element_text(size=14,face="bold"),legend.key=element_blank
())+
  theme(aspect.ratio = 1/sqrt(2))+ #fix plot size
  theme(axis.text=element_text(size=12), #fix axis font size
        axis.title=element_text(size=14,face="bold"))+ #fix axis title font size
  theme(strip.text.x = element_text(size = 12,face="bold"))+
  theme(panel.grid.major.y = element_line(colour = "#111111",size=0.2), #fix grid display
        panel.grid.minor.y = element_blank(), #fix grid display
        panel.grid.major.x = element_blank(),
        axis.title.x= element_blank(),
        panel.grid.minor = element_blank(),
        strip.background = element_rect(fill = 'transparent'),
        panel.background = element_blank(), axis.line = element_line(colour = "black"))
MVPAplot35scr
ggsave(
  "Figure5b.pdf",
  plot = MVPAplot35scr,
  unit=c("mm"),
  width = gwidth,
  height = gheight,
  scale=3
)
```

Supplement 7: Additional control conditions:

Control comparisons: Effects of scrambled vs. normal picture viewing

In addition to pictures with emotional content, participants were shown scrambled pictures. This control condition was used to explore potential oxytocin effects on attention. For this purpose, the analyses above were repeated, replacing the factor "Emotion" by the factor "Scrambled" (levels: scrambled pictures vs. pictures).

The main effect of scrambled vs. clear pictures was only marginally significant for Intensity Ratings ($F[1, 29] = 4.02$, $p = .054$, see: Fig. S5.1). Viewing normal pictures compared to scrambled pictures decreased intensity ratings by -1.28 points (VAS, 95 %CI [-2.58 0.03], $\beta = -0.05$) across medication and temperature conditions. The effect of picture scrambling did not reach statistical significance for Unpleasantness Ratings ($F[1, 29] = 1.32$, $p = .26$), despite a similar direction of effect (-0.83 points VAS, 95 %CI [-2.31 0.65], $\beta = -0.03$). Similarly, MHT estimates were affected by factor Scrambled ($F[1, 29.0] = 5.50$, $p = .018$, Fig. S5.2): Viewing normal compared to scrambled pictures decreased MHT by -0.09 units, 95 %CI [-0.16, -0.02], $\beta = -0.15$), the size and direction of effect corresponding approximately with the effect of emotional picture viewing on ratings. Factor scrambled did not show any interaction with factors oxytocin or temperature for any variable ($p>.41$). Together, these results indicate that viewing pictures compared to scrambled pictures slightly decreased ratings and activity of whole-brain correlates of temperature intensity processing.

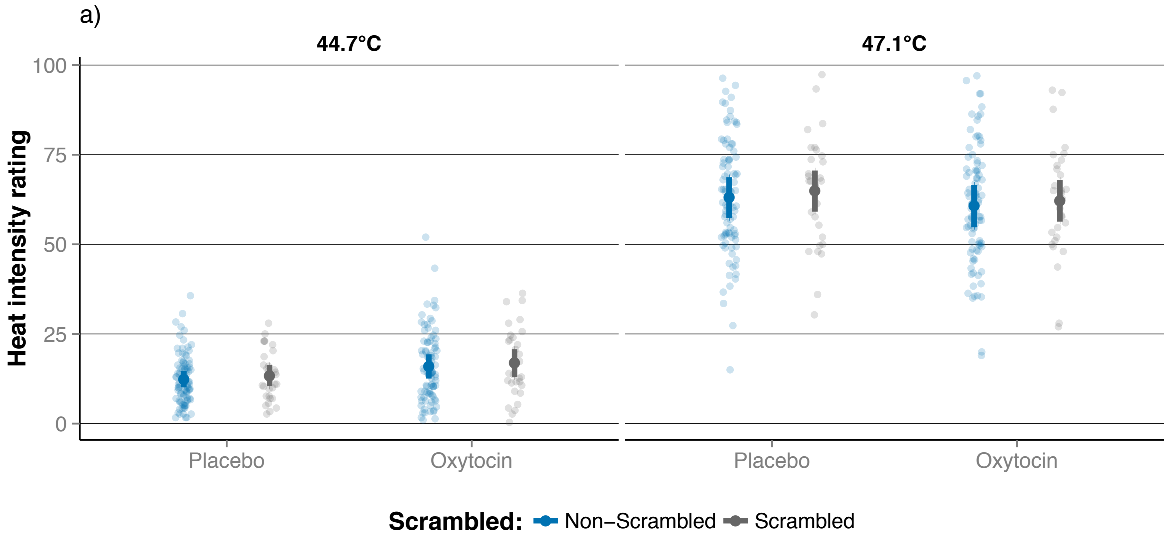

Fig. S5.1: Rating results — normal pictures versus scrambled control

Effects of oxytocin, temperature and emotional picture valence on intensity ratings. Figures depict estimated marginal means and 95% Confidence Intervals (CI) as estimated by mixed model analysis on top of single subject data.

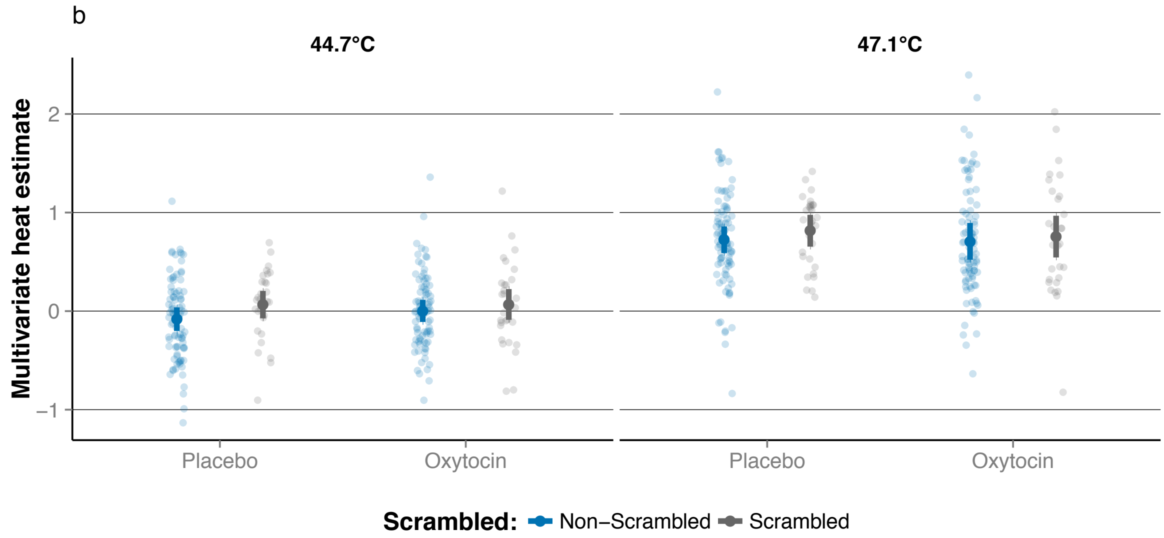

Fig. S5.2: Multi-voxel heat temperature results — normal pictures versus scrambled control

Effects of oxytocin, temperature and emotional picture valence on b) multi-voxel heat

temperature (MHT). Figures depict estimated marginal means and 95% Confidence Intervals (CI) as estimated by mixed model analysis on top of single subject data.

Baseline temperature control:

We examined the effects of picture-viewing on MHT at baseline temperature (35°C), to determine whether they were affected by visual stimulation per se. The majority of intensity and unpleasantness ratings at baseline temperature equaled zero and were therefore not analyzed. For MHT, there was no significant main effect of oxytocin ($p = .72$), emotional valence ($p = .17$) and no interaction between the two ($p = .90$, Fig. S5.3) at baseline temperatures. However, negative picture viewing tended to decrease MHT (-0.17 units, 95 %CI [-0.36, 0.01], $\beta = -0.29$), the size and direction of effect being equivalent to the effect observed at higher temperatures.

MHT estimates at 35 °C (Fig. S5.4) did not show a significant main effect of factor scrambled ($F[1, 29.0] = 3.08$, $p = .090$), or any interaction with medication ($p = .332$). Again, effect sizes suggested that viewing normal compared to scrambled pictures decrease MHT (-0.11 units, 95 %CI [-0.24, 0.02], $\beta = -0.25$), similar to what was observed at higher temperatures. These results suggest that emotional picture valence and picture stimulation per se may have affected MHT independent of temperature.

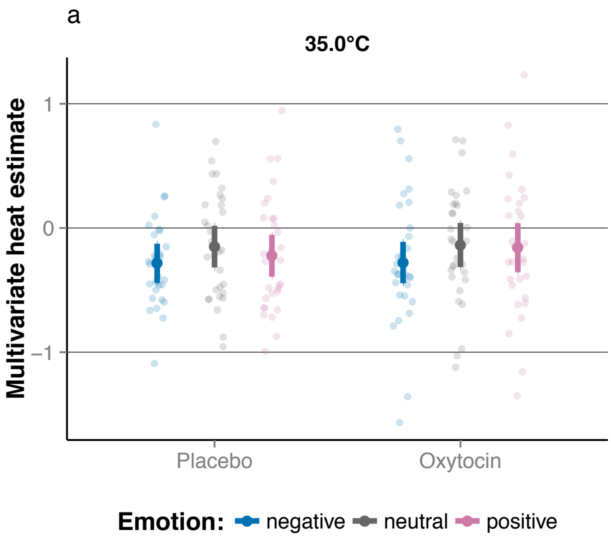

Fig. S5.3: Multi-voxel heat temperature results — baseline temperature control and emotional pictures

Effects of oxytocin, temperature and emotional picture valence on multi-voxel heat temperature (MHT). Figures depict estimated marginal means and 95% Confidence Intervals (CI) as estimated by mixed model analysis on top of single subject data.

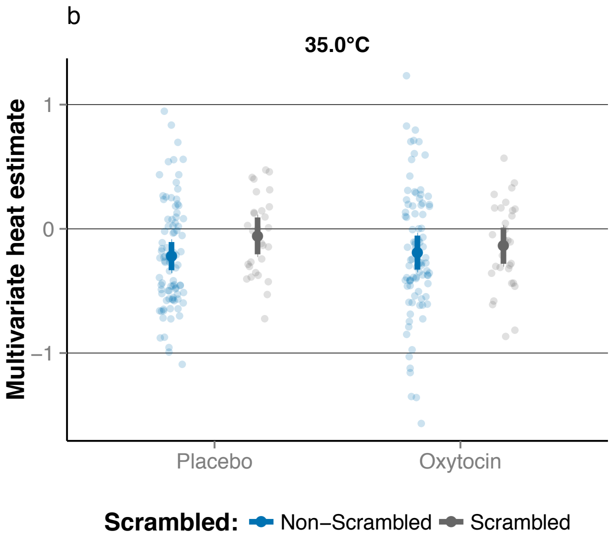

Fig. S5.4: Multi-voxel heat temperature results — baseline temperature control and

scrambled pictures

Effects of oxytocin, temperature, as well as picture viewing versus scrambled picture viewing on multi-voxel heat temperature (MHT). Figures depict estimated marginal means and 95% Confidence Intervals (CI) as estimated by mixed model analysis on top of single subject data.

Pain Yes/No ratings:

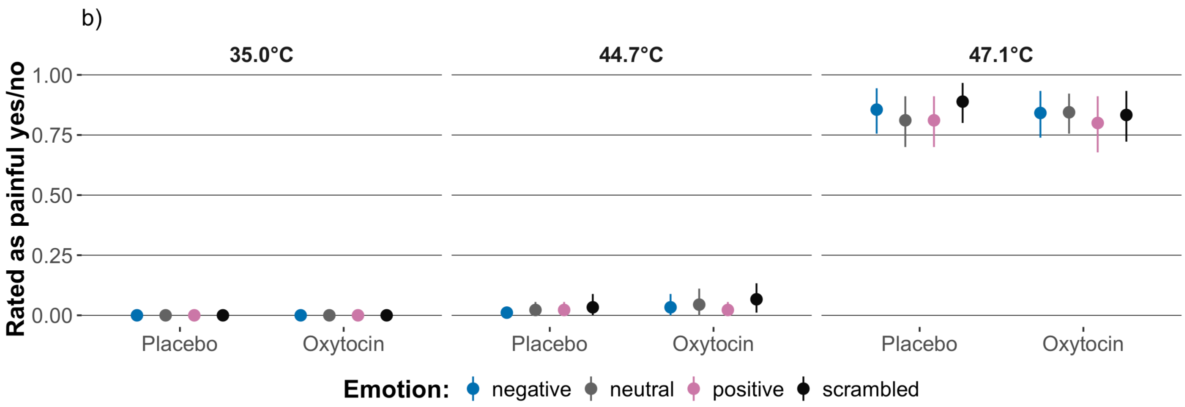

Fig. S5.5: Categorical pain rating results

Participants were required to categorize stimuli as painful or not in addition to heat intensity and unpleasantness ratings. Figure depicts the ratio of painful to non-painful ratings with boot-strapped 95% Confidence Intervals (CI).

Categorical pain ratings were obtained as a control measure, in order to verify that the temperature conditions chosen qualified as non-noxious and noxious, respectively. Only 3.1% of all stimuli at 44.7°C were classified as painful across all conditions, whereas 83.6% of all stimuli were classified as painful at 47.1°C. Not a single baseline temperature stimulus (35.0°C) was rated as painful (see: Supplement 5). It should be noted that the hot, non-noxious temperature level (44.7°C) is slightly above the heat pain threshold that was determined for un-stimulated skin using the method of limits [34]. However, due to the pre-

conditioning of the skin with training stimuli and the repeated tonic stimulation, tonic 14-second stimuli at 44.7°C are perceived as non-noxious — likely due to de-sensitizing effects.

References:

[1] Cohen J. A power primer. Psychol. Bull. 1992;112:155–159.

[2] Colden A, Bruder M, Manstead ASR. Human content in affect-inducing stimuli: A secondary analysis of the international affective picture system. Motiv. Emot. 2008;32:260–269.

[3] Dunlap WP, Cortina JM, Vaslow JB, Burke MJ. Meta-analysis of experiments with matched groups or repeated measures designs. Psychol. Methods 1996;1:170–177.

[4] Horjales-Araujo E, Demontis D, Lund EK, Vase L, Finnerup NB, Børglum AD, Jensen TS, Svensson P. Emotional modulation of muscle pain is associated with polymorphisms in the serotonin transporter gene. Pain 2013;154:1469–76.

[5] Kamping S, Bomba IC, Kanske P, Diesch E, Flor H. Deficient modulation of pain by a positive emotional context in fibromyalgia patients. Pain 2013;154:1846–55.

[6] Rainer G, Augath M, Trinath T, Logothetis NK. The Effect of Image Scrambling on Visual Cortical BOLD Activity in the Anesthetized Monkey. Neuroimage 2002;16:607–616.

[7] Rasch B, Friese M, Hofmann W, Naumann E. Quantitative Methoden 2. Einführung in die Statistik für Psychologen und Sozialwissenschaftler. 3rd ed. Heidelberg: SpringerMedizin, 2010.

[8] Rhudy JL, Williams AE, McCabe KM, Russell JL, Maynard LJ. Emotional control of nociceptive reactions (ECON): do affective valence and arousal play a role? Pain 2008;136:250–61.

[9] Roy M, Lebuis A, Peretz I, Rainville P. The modulation of pain by attention and emotion: a dissociation of perceptual and spinal nociceptive processes. Eur. J. Pain 2011;15:641.e1–10.

[10] Tibshirani R. Regression Shrinkage and Selection via the Lasso. J. R. Stat. Soc. Ser. B 1996;58:267–288.

[11] Varma S, Simon R. Bias in error estimation when using cross-validation for model selection. BMC Bioinformatics 2006;7:91.

[12] Wager TD, Atlas LY, Lindquist M a, Roy M, Woo C-W, Kross E. An fMRI-based neurologic signature of physical pain. N. Engl. J. Med. 2013;368:1388–97.

[13] Zunhammer M, Geis S, Busch V, Greenlee MW, Eichhammer P. Effects of Intranasal Oxytocin on Thermal Pain in Healthy Men. Psychosom. Med. 2015;77:156–166.
